# Comparison of PET/CT and MRI in the Diagnosis of Bone Metastasis in Prostate Cancer Patients: A Network Analysis of Diagnostic Studies

**DOI:** 10.3389/fonc.2021.736654

**Published:** 2021-10-04

**Authors:** Fanxiao Liu, Jinlei Dong, Yelong Shen, Canhua Yun, Ruixiao Wang, Ganggang Wang, Jiyang Tan, Tao Wang, Qun Yao, Bomin Wang, Lianxin Li, Jingyi Mi, Dongsheng Zhou, Fei Xiong

**Affiliations:** ^1^ Department of Orthopaedics, Shandong Provincial Hospital Affiliated to Shandong First Medical University, Jinan, China; ^2^ Department of Medical Imaging, Shandong Provincial Hospital Affiliated to Shandong First Medical University, Jinan, China; ^3^ Department of Nuclear Medicine, The Second Hospital of Shandong University, Jinan, China; ^4^ Department of Urology Surgery, University Hospital of Munich, Ludwig-Maximilians-University, Munich, Germany; ^5^ Department of Urology Surgery, Shandong Provincial Hospital Affiliated to Shandong University, Jinan, China; ^6^ Department of Sports Medicine, Wuxi 9th People’s Hospital Affiliated to Soochow University, Wuxi, China; ^7^ Orthopaedic Institute, Medical College, Soochow University, Suzhou, China

**Keywords:** prostate cancer, bone metastasis, diagnostic study, network meta-analysis, MRI, PET/CT

## Abstract

**Background:**

Accurate diagnosis of bone metastasis status of prostate cancer (PCa) is becoming increasingly more important in guiding local and systemic treatment. Positron emission tomography/computed tomography (PET/CT) and magnetic resonance imaging (MRI) have increasingly been utilized globally to assess the bone metastases in PCa. Our meta-analysis was a high-volume series in which the utility of PET/CT with different radioligands was compared to MRI with different parameters in this setting.

**Materials and Methods:**

Three databases, including Medline, Embase, and Cochrane Library, were searched to retrieve original trials from their inception to August 31, 2019 according to the Preferred Reporting Items for Systematic Review and Meta-analysis (PRISMA) statement. The methodological quality of the included studies was assessed by two independent investigators utilizing Quality Assessment of Diagnostic Accuracy Studies (QUADAS-2). A Bayesian network meta-analysis was performed using an arm-based model. Absolute sensitivity and specificity, relative sensitivity and specificity, diagnostic odds ratio (DOR), and superiority index, and their associated 95% confidence intervals (CI) were used to assess the diagnostic value.

**Results:**

Forty-five studies with 2,843 patients and 4,263 lesions were identified. Network meta-analysis reveals that 68Ga-labeled prostate membrane antigen (68Ga-PSMA) PET/CT has the highest superiority index (7.30) with the sensitivity of 0.91 and specificity of 0.99, followed by 18F-NaF, 11C-choline, 18F-choline, 18F-fludeoxyglucose (FDG), and 18F-fluciclovine PET/CT. The use of high magnetic field strength, multisequence, diffusion-weighted imaging (DWI), and more imaging planes will increase the diagnostic value of MRI for the detection of bone metastasis in prostate cancer patients. Where available, 3.0-T high-quality MRI approaches 68Ga-PSMA PET/CT was performed in the detection of bone metastasis on patient-based level (sensitivity, 0.94 *vs.* 0.91; specificity, 0.94 *vs.* 0.96; superiority index, 4.43 *vs.* 4.56).

**Conclusions:**

68Ga-PSMA PET/CT is recommended for the diagnosis of bone metastasis in prostate cancer patients. Where available, 3.0-T high-quality MRI approaches 68Ga-PSMA PET/CT should be performed in the detection of bone metastasis.

## 1 Introduction

Prostate cancer (PCa) is the second most frequently diagnosed cancer and the fifth leading cause of cancer death in men according to global cancer statistics in 2018 (1). Although the 5-year survival rate is fairly high, the common cause of death is bone metastasis (2–5), which is the second most site of metastases in PCa (6, 7). Patients with an early diagnosis of localized disease may benefit from radical localized curative treatment (8), but patients who suffer from bone metastasis may be only eligible for hormone therapy or chemotherapy (9, 10). Hence, assessment of bone metastasis status, especially the early detection, is an important issue in the management of PCa. To follow and quantify the metastasis extent, which is an independent prognostic factor (11), the use of non-invasive imaging modalities is essential (12).

For decades, European and US guidelines recommend bone scintigraphy (BS) for bone metastasis diagnosis, which, if necessary, can be complemented by targeted X-rays (TXR) (13, 14). This BS/TXR association is imperfect, which strikingly lacks diagnostic specificity (15, 16), although the use of single-photon emission computed tomography (SPECT) improves the resolution (8, 17). Equivocal imaging results of BS are required to be determined by the additional use of magnetic resonance imaging (MRI); however, this multiple approach can add to the cost and become inconvenient for patients (18). The accuracy of MRI to detect bone metastasis has been highlighted for almost 30 years, and the superiority than BS has also been repeatedly suggested (18–21). A meta-analysis conducted by Woo et al. demonstrated the excellent diagnostic performance of MRI for the detection of bone metastasis in PCa (22). The development of new technology, such as diffusion-weighted imaging (DWI) (23), and the application of whole-body MRI (WB-MRI) may further expand the potential of MRI (20, 24, 25). However, the use of MRI for one-step cancer tumor–node–metastasis (TMN) staging is often presented as not feasible due to costs and the limited study validating this modality (26).

In recent years, positron emission tomography/computed tomography (PET/CT) has emerged as a promising molecular imaging tool in the diagnosis, staging, restaging, and therapeutic evaluation of several malignancies, as PET provides metabolic information and morphological imaging techniques offer anatomical data (27, 28). 18F-fluorodeoxyglucose (18F-FDG) is the most widely used PET-imaging agent in oncology detection; however, the low glycolytic rate of most skeletal metastases in PCa and the influence of bladder activity limit the sensitivity for clinical detection (7). The European Nuclear Medical Association recommended PET/CT in their guidelines for bone imaging in 2015 (29), which can show areas of altered osteogenic activity by using 18F-sodium fluoride (18F-NaF), a bone-specific radiotracer (30). Additionally, 11C- or 18F-choline are designed to target tumor cells directly (31), and the European Association of Urology (EAU) once suggested referring patients for 11C- or 18F-choline PET/CT when the prostate-specific antigen (PSA) increases >1 ng/ml, and the result is expected to change patient management (32). Over the last 5 years, 68Ga-labeled prostate-specific membrane antigen (68Ga-PSMA) PET/CT has gained widespread use to assess PCa (33, 34), which could identify metastatic lesions in lymph node, bone, and soft tissue at low PSA levels (33–35). Therefore, 68Ga-PSMA PET/CT is regarded as a more specific modality for diagnosing osseous metastases in PCa (36).

Despite the increasing numbers of studies regarding PET/CT and MRI in the diagnosis procedure for bone metastases in PCa, the effectiveness of these two modalities still remains no consensus. Zhou et al. (37) compared PET/CT and MRI; the final conclusion was very general because of the limitation of traditional meta-methods, which could not directly compare the PET/CT using different radioligands and MRI with different parameters. Recently, Nyaga et al. (38) developed a Bayesian network meta-analysis using an arm-based model based on the assumption that the missing arms occur at random. This method has been applied in several studies (39, 40) because it could allow analysis of the variability in the accuracy of multiple tests within and between studies simultaneously (41).

The arm-based model is more appealing than traditional meta-analysis and the contrast-based model since the former not only permits more straightforward interpretation of the parameters, making use of all available data and yielding shorter credible intervals, but also provides more natural variance–covariance matrix structures. We adopted this model, which makes our results more convincing.

Thus, the primary aim of our meta-analysis is to compare the diagnostic accuracy of PET/CT and MRI in detecting bone metastases in PCa on a per-patient and per-lesion basis, respectively. Additionally, thanks to the establishment of the network meta-analysis, a direct comparison is performed between PET/CT with different radioligands and MRI with different magnet field strengths, coverage, and parameters to provide better evidence-based advice to physicians. The hypothesis is that the multiparametric MRI equipped with high magnet field strength is an appropriate modality for the diagnosis of bone metastasis in PCa.

## 2 Materials and Methods

### 2.1 Protocol and Guidance

This network meta-analysis was conducted in accordance with the Preferred Reporting Items for a Systematic Review and Meta-analysis of Diagnostic Test Accuracy Studies (PRISMA-DTA) (42) statement and PRISMA (PROSPERO registration number CRD42020148865).

### 2.2 Inclusion Criteria

The included studies should meet all of the following inclusion criteria: clinical trials evaluating the diagnostic value of PET/CT or/and MRI for bone metastasis in prostate cancer patients; articles published providing data to calculate diagnostic parameters, including true positive (TP), false positive (FP), false negative (FN), and true negative (TN); and studies having a conclusive anatomical or morphological verification standard to prove or disprove the imaging study result, such as pathological examination or clinical confirmation.

### 2.3 Exclusion Criteria

We excluded studies if they were commentaries, letters, case reports, reviews or non-full-text studies; if all the participants were prostate patients with bone metastasis; and if they lack of the conclusive anatomical or morphological verification standard to prove or disprove the imaging study result, such as pathological examination or clinical confirmation.

### 2.4 Outcomes

The outcomes are the absolute sensitivity and specificity, relative sensitivity and specificity, diagnostic odds ratio (DOR), and superiority index of PET/CT with numerous tracers and MRI with different parameters for the diagnosis of bone metastasis in prostate cancer patients. **Supplementary Table S1** shows the definition of these outcomes.

### 2.5 Search Strategy

Three databases, including Medline, Embase, and Cochrane Library, were searched to retrieve original trials from their inception to August 31, 2019. We also searched ClinicalTrials.gov and the World Health Organization International Clinical Trials Registry Platform to identify ongoing or unpublished eligible trials. To maximize the search for relevant articles, a manual search of the references listed in all included trials and systematic reviews was performed to retrieve any relevant articles that were not listed in the databases. **Supplementary Table S2** shows the search strategy.

### 2.6 Study Selection

After removal of duplicates, two investigators performed a blind systematic screening for all titles and abstracts in duplicate. Then, the full texts of the remainders were downloaded to confirm their eligibility based on the above criteria. To maximize the sensitivity of the screen, disagreements at the title and abstract stages were resolved by automatic inclusion, whereas discrepancies at the full-text stage were resolved by consensus with input from a senior third investigator.

### 2.7 Data Collection Process

Two independent investigators used a predesigned Microsoft Excel spreadsheet (Version 2013, Microsoft, Redmond, WA, USA) to extract basic information from the included studies. The diagnostic data (TPs, FPs, FNs, and TNs) were extracted or calculated using the following methods, which are presented in **Supplementary Table S3**. The spreadsheets were combined, and each investigator checked a random selection of the other’s entries for quality control. Any discrepancies were resolved by consensus.

### 2.8 Quality Assessment of Included Studies

The methodological quality of the included studies was assessed by two independent investigators utilizing Quality Assessment of Diagnostic Accuracy Studies (QUADAS-2) tool, which comprised of four key domains (patient selection, index test, reference standard, and flow and timing). Detailed information is shown in **Supplementary Table S4**.

### 2.9 Data Synthesis

A Bayesian network meta-analysis using an arm-based model, developed by Nyaga et al., was performed by running three chains in parallel until there is convergence. We used absolute sensitivity and specificity, relative sensitivity and specificity, DOR, and superiority index and their associated 95% confidence intervals (CI) to assess the diagnostic value of PET/CT and MRI. To assess the relative performance of diagnostic tests, the definitions of superior, inferior, equal, and not comparable were drawn. A diagnostic test, which is pairwise superior to a relatively large number of other tests and pairwise inferior to relatively few other tests, should have a high superiority value and be ranked higher than those tests that do not perform as well. In this network meta-analysis, superiority index was pooled to quantify rank probabilities of a PET/CT and MRI. All network meta-analyses were performed using R (v3.4.3; Comprehensive R Archive Network). **Supplementary Table S5** shows the main information of implementation process, software’s packages, and models.

#### 2.9.1 Subgroup Analyses

For different tracers of PET/CT, we performed several subgroup analyses according to clinical settings of prostate cancer (new diagnoses, mixed, and treated), number of patients (<50 and ≥50), patient age (60–70 and > 70), continent of origin (Europe and others), study design (prospective and retrospective), and methods of imaging analyses (visual and semiquantitative evaluation). For the parameters of MRI, we performed several subgroup analyses according to magnetic field strength (1.5 and 3.0 T), the number of sequences (single sequences, or ≥ multisequences), whether using DWI or not, number of imaging planes (1 or ≥2), and MRI coverage (pelvis skeleton, axial skeleton, or whole-body skeleton).

#### 2.9.2 Sensitivity Analyses

The sensitivity analyses were performed by deleting studies involving only one diagnostic test for detecting bone metastasis, studies with low a QUADAS-2 score, studies with the maximum cases, studies with the minimum cases, and studies published before 2010.

### 2.10 Patient and Public Involvement

No patients were involved in setting the research question or the outcome measures, nor were they involved in developing plans for design or implementation of the study. No patients were asked to advise on interpretation or writing up of results. There are no plans to disseminate the results of the research to study participants or the relevant patient community. It was not evaluated whether the studies included in the review had any patient involvement.

## 3 Results

### 3.1 Eligible Studies and Study Characteristics

**Figure 1:**
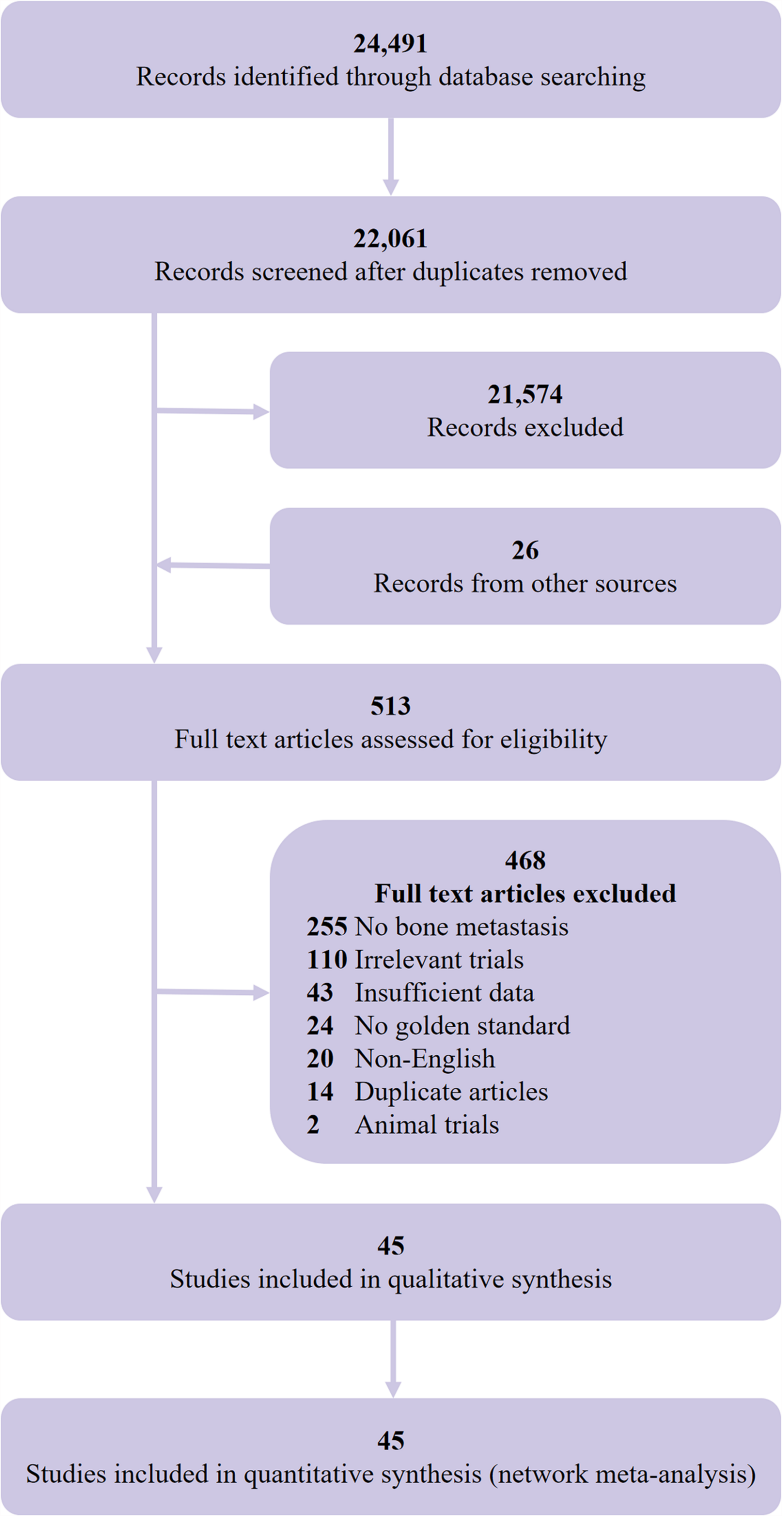
Selection flow chart for studies included in the network meta-analysis.

We initially identified 24,491 records and included 45 eligible studies (8, 43–86) in the final network meta-analysis (**Figure 1**). The studies comprised 2,843 participants (4,263 lesions), with 978 bone metastasis patients (2,186 lesions), and a total of 32 studies assessed the diagnostic value of PET/CT and 21 studies for MRI. **Table 1** shows the main characteristics of the included studies. **Supplementary Tables S6**, **S7** show the main technical parameters of MRI and PET/CT, respectively. **Supplementary Tables S8**, **9** show the diagnostic data of each included studies on patient- and lesion-based level, respectively. **Supplementary Table S10** shows the quality assessment of the included studies. Four (8.9%), 11 (24.4%), 18 (40.0%), 11 (4.5%), and 1 (2.2%) studies scored 11, 10, 9, 8, and 7, respectively.

**Table 1:** Main characteristics of the included studies.

Study, year	Country	No. of patients			Clinical setting	Age (years)		PSA (ng/ml)		Clinical T stage	Gleason score		Type of study	Inclusion interval
		Total (n)	Metastasis		New or treated	Mean ± SD	Range	Mean ± SD	Range	Range	Median	Range		
			n	%										
Even-Sapir et al. (8)	Israel	44	23	52.27	Mixed	71.6 ± 8.8	NR	NR	≥20	NR	NR	≥8	P	NR
Eschmann et al. (43)	Germany	44	44	100.00	Mixed	Median 64.1	51–79	Median 5.4	0.15–200	T1–T4	NR	NR	R	11.2004–01.2006
Lecouvet et al. (44)	USA	66	41	62.12	Mixed	74	46–85	NR	NR	NR	NR	NR	P	NR
Nemeth et al. (45)	USA	8	7	87.50	NR	NR	NR	NR	NR	NR	NR	NR	R	1998–2004
Beheshti et al. (46)	Austria	38	NR	–	Mixed	69 ± 8	NR	56 ± 64	NR	NR	NR	NR	P	NR
Beheshti et al. (6)	Austria	70	NR	–	Mixed	68 ± 7	NR	NR	≥10	NR	NR	≥7	P	NR
Venkitaraman et al. (47, 48)	UK	99	14	14.14	Newly diagnosed	Median 66	44–83	Median 26.5	2–1600	NR	7	6–10	P	12.2001–12.2005
Venkitaraman et al. (47, 48)^#^	UK	39	10	25.64	Newly diagnosed	Median 65	54–82	Median 34	5–1300	T1–T4	8	5–9	P	12.2001–07.2004
Fuccio et al. (50)	Italy	25	22	88.00	Treated	70.2	58–80	Median 6.3	0.2–37.7	T2N0/Nx M0-T4N1M0	7	6–9	R	NR
Iagaru et al. (51)	USA	18	9	50.00	Mixed	NR	NR	NR	NR	NR	NR	NR	P	09.2007–12.2010
Langsteger et al. (52)	Austria	40	22	55.00	Mixed	66	51–82	NR	0.38–617	NR	NR	4–9	P	01.2003–12.2009
Bortot et al. (53)	Brazil	9	2	22.22	NR	67.6	56–82	NR	NR	NR	NR	NR	P	NR
Jadvar et a (54)	USA	37	14	37.84	Treated	Median 71.1	53.5–86.9	Median 3.2	0.5–40.2	T1c–T3	NR	NR	P	22.09.2010–23.06.2011
Lecouvet et al. (55)	Belgium	100	51	51.00	Mixed	69	53–88	32	12–78	≥T3b	NR	≥8	P	03.2007–03.2010
Mosavi et al. (56)	Sweden	49	5	10.20	Newly diagnosed	Median 67	57–80	Median 14	1.3–950	T1c–T4	9	8–10	P	01.2009–03.2011
Picchio et al. (57)	Italy	78	27	34.62	Treated	69	47–82	21.1	0.2–500.0	T2N0–T4N0	NR	NR	R	03.2005–02.2010
Takesh et al. (58)	Germany	37	18	48.65	Treated	69 ± 7	NR	2.6	0.3–21	NR	7	3–9	R	NR
Damle et al. (59)	India	49	32	65.31	Mixed	65	50–84	NR	NR	T3/T4	NR	8–10	P	NR
Kitajima et al. (60)	USA	95	16	16.84	Treated	65.7	49–87	Median 2.5	0.58–68.3	T2N0–any T N1	7	2–10	R	12.2011–01.2013
Pasoglou et al. (61)	Belgium	30	9	30.00	Treated	Median 62.5	51.0–92.0	Median 30	1.7–4612.0	cT2–cT4	7.8	6–9	P	NR
Piccardo et al. (62)	Italy	21	6	28.57	Treated	77.2 ± 5.1	70–85	5.8 ± 3.4	2.2–13.4	NR	8	7–9	P	NR
Poulsen et al. (63)	Denmark	50	NR	–	Treated	73 ± 8.6	53–94	Median 84	4–5740	NR	7	5–10	P	05.2009–03.2012
Evangelista et al. (64)	Italy	48	11	22.92	Newly diagnosed	70	49–86	38.34 ± 90.12	2.80–581.0	T2–T4	NR	6–10	R	04.2010-04.2013
Pasoglou et al. (65)	Belgium	30	10	33.33	Mixed	69	NR	31 ± 28	NR	NR	NR	NR	P	02.2012–12.2012
Sampath et al. (66)	USA	38	22	57.89	NR	NR	NR	NR	NR	NR	NR	NR	R	09.2007–07.2013
Wieder et al. (67)	Germany	57	50	87.72	Treated	Median 86	54–80	29.9	1–670	NR	8	6–9	P	NR
Barchetti et al. (68)	Italy	152	70	46.05	Treated	NR	53–88	NR	NR	NR	NR	≥7	P	09.2011–01.2014
Conde-Moreno et al. (69)	Spain	35	17	48.57	Treated	Median 70 ± 6.77	52–80	Median 12	4.54–75.86	T1N0M0–T4N0M0	7	5–9	P	01.2014–03.2015
Nanni et al. (70)	Italy	89	6	6.74	Mixed	69	55–83	6.99	0.20–20.72	T1N0/Nx–T3N0/Nx	NR	NR	P	NR
Woo et al. (71)	Korea	308	21	6.82	Newly diagnosed	68.5 ± 7.8	38–91	30.9	1.2–955.5	NR	7	6–10	R	01.2013-12.2013
Yi et al. (72)	China	26	12	46.15	Mixed	72.2	60–88	NR	≥20	T2–T4	NR	8–10	P	08.2010–11.2014
Fonager et al. (73)	Denmark	37	27	72.97	Mixed	71	46–87	Median 180	53–9708	T1–T4	9	7–10	R	02.2014–12.2015
Huysse et al. (74)	Belgium	64	62	96.88	Treated	NR	NR	NR	NR	NR	NR	NR	P	NR
Janssen et al. (75)	Germany	54	29	53.70	Mixed	69.6 ± 6.5	NR	38.4 ± 77.9	NR	NR	NR	NR	P	NR
Kitajima et al. (76)	Japan	21	11	52.38	Mixed	70.6 ± 10.8	47–90	342.9	0.2–5916	NR	NR	NR	P	01.2015–01.2017
Vargas et al. (77)	USA	228	57	25.00	Newly diagnosed	Median 63	36-83	Median 6.3	0.4–222	T1c–T4	7	6–≥8	R	01.2000–06.2014
Wondergem et al. (78)	Netherlands	104	61	58.65	Mixed	74.9	49–93	Median 88.7	2.5–13500	T1–T4	9	6–10	R	01.2011–04.2012
Dyrberg et al. (79)	Denmark	55	20	36.36	Newly diagnosed	75 ± 9	54–91	85	5–1000	NR	8	6–10	P	05.2016–06.2017
Kawanaka et al. (80)	Japan	30	17	56.67	Treated	71.3 ± 9.0	47–90	65.2 ± 177.4	0.23–946	NR	NR	NR	R	01.2015–07.2017
Larbi et al. (81)	Belgium	50	37	74.00	Newly diagnosed	67 ± 10	59–87	NR	≥20	NR	NR	≥ 8	R	01.2015–12.2015
Lengana et al. (82)	South Africa	113	26	23.01	Mixed	66.65	43–88	NR	NR	NR	NR	NR	P	NR
Zacho et al. (83)	Denmark	68	10	14.71	Treated	67.2	47–80	NR	0.2–11	M0–M1	7	5–9	P	NR
Chen et al. (84)	USA	106	14	13.21	Mixed	Median 70	47–80	Median 1.3	0–61	NR	8	6–10	R	01.2017–01.2018
Johnston et al. (85)	UK	56	5	8.93	Newly diagnosed	67.9	57.9–84.4	Median 20.05	10.07–61.20	NR	7	6–10	P	07.2012–11.2015
Uslu–besli et al. (86)	Turkey	28	11	39.29	Mixed	67.3 ± 7.4	49–82	25.49 ± 32.7	0.5–125.1	NR	7	6–9	R	03.2015-03.2016

SD, Standard deviation; NR, Not reported; PSA, Prostate specific antigen; P, Prospective; R, Retrospective. ^#^Represents different study.

### 3.2 Diagnostic Value of PET/CT on Per-Patient Analysis

**Figure 2:**
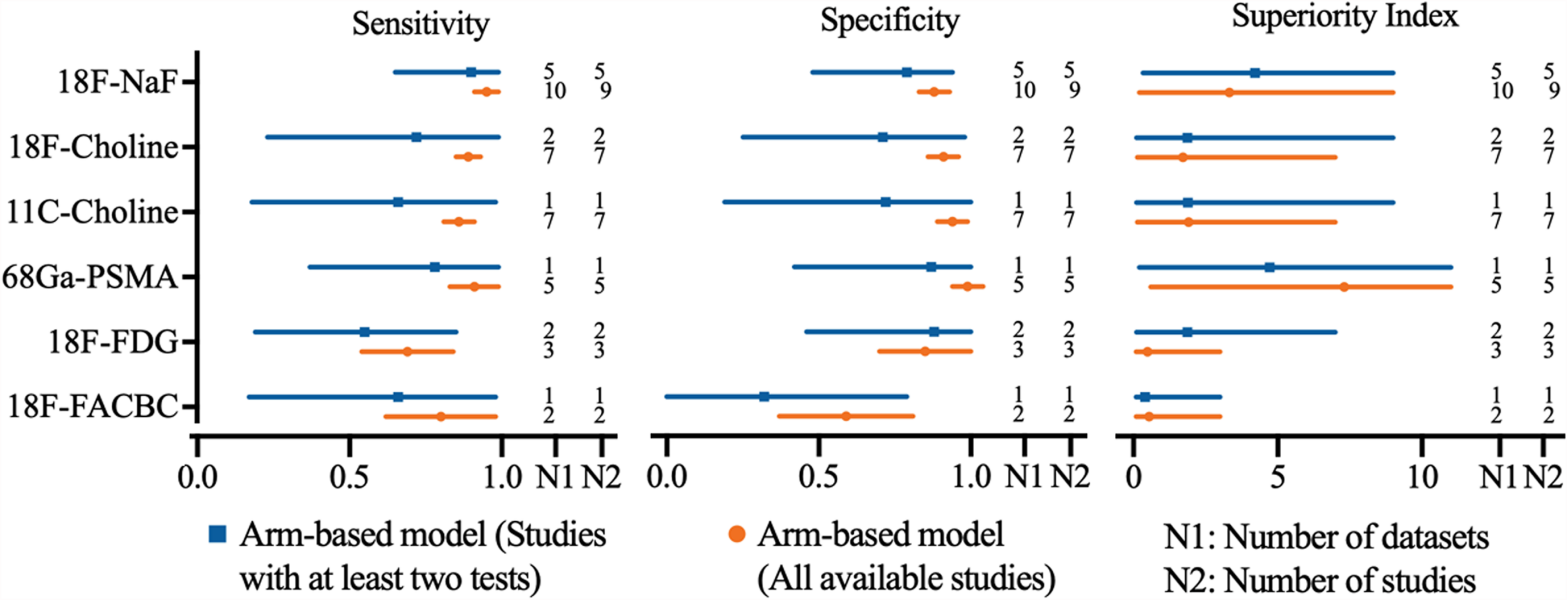
Network meta-analysis results including sensitivity, specificity, and superiority index values of PET/CT with six commonly used tracers for the detection of bone metastasis in prostate cancer patients. Sensitivity, specificity, and superiority index are reported as mean (range) unless otherwise indicated. PET/CT, positron emission tomography/computed tomography; NaF, sodium fluoride; PSMA, prostate membrane antigen; FDG, fludeoxyglucose; FACBC, fluciclovine.

Regarding the tracers of the included studies, nine used 18F-NaF, seven selected 18F-choline, seven applied 11C-choline, five used 68Ga-PSMA, three selected 18F-FDG, and two applied 18F-FACBC. As shown in **Figure 2**, 18F-NaF PET/CT has the highest sensitivity of 0.95 (95% CI, 0.91–0.99), followed closely by 68Ga-PSMA and 18F-choline PET/CT; 68Ga-PSMA PET/CT has the highest specificity of 0.99 (95% CI, 0.94-1.04), followed closely by 11C-choline and 18F-choline PET/CT. Network meta-analysis demonstrated that 68Ga-PSMA PET/CT has the highest diagnostic value with the highest superiority index of 7.30 (95% CI, 0.60–11.00), followed closely by 18F-NaF (3.33; 95% CI, 0.20–9.00), 11C-choline, 18F-choline, 18F-FACBC, and 18F-FDG PET/CT (**Table 2**).

**Table 2:** The network meta-analysis results of PET/CT with different tracers to detect bone metastasis in PCa.

Test	Absolute Sensitivity	Absolute Specificity	Diagnostic OR [Rank]	SuperiorityIndex [Rank]		Relative Sensitivity	Relative Specificity	Datasets, n	Studies, n
18F-NaF	0.95 (0.91–0.99)	0.88 (0.83–0.93)	248.15 (34.14–799.75) [2]	3.33 (0.20–9.00) [2]	1.00 (1.00–1.00)		1.00 (1.00–1.00)	10	9
18F-Choline	0.89 (0.85–0.93)	0.91 (0.86–0.96)	123.18 (21.09–361.86) [4]	1.71 (0.14–7.00) [4]	0.94 (0.88–1.00)		1.03 (0.95–1.11)	7	7
11C-Choline	0.86 (0.81–0.91)	0.94 (0.89–0.99)	208.88 (21.37–780.88) [3]	1.92 (0.14–7.00) [3]	0.91 (0.84–0.98)		1.07 (0.98–1.16)	7	7
68Ga-PSMA	0.91 (0.83–0.99)	0.99 (0.94–1.04)	3379817.37 (49.99–5941029.19) [1]	7.30 (0.60–11.00) [1]	0.96 (0.87–1.05)		1.12 (1.04–1.20)	5	5
18F-FDG	0.69 (0.54–0.84)	0.85 (0.70–1.00)	81.17 (1.35–497.30) [6]	0.49 (0.09–3.00) [6]	0.73 (0.57–0.89)		0.96 (0.78–1.14)	3	3
18F-FACBC	0.80 (0.62–0.98)	0.59 (0.37–0.81)	92.40 (0.40–598.84) [5]	0.55 (0.09–3.00) [5]	0.84 (0.65–1.03)		0.67 (0.42–0.92)	2	2

Data are reported as mean (range) unless otherwise indicated.

PET/CT, positron emission tomography/computed tomography; PCa, prostate cancer; NaF, sodium fluoride; PSMA, prostate membrane antigen; FDG, fludeoxyglucose; FACBC, fluciclovine; CI, credible interval; OR, odds ratio.

#### 3.2.1 Sensitivity Analysis

The sensitivity analyses were performed by deleting studies involving only one diagnostic test for detecting bone metastasis, studies with low QUADAS-2 score, studies with the maximum cases, studies with the minimum cases, and studies published before 2010, and the results were stable (**Supplementary Tables S11**–**S15**).

#### 3.2.2 Subgroup Analysis

The subgroup analyses were performed according to clinical settings of prostate cancer (newly diagnoses, mixed, and treated), number of patients (<50 and ≥50), patient age (60–70 and >70), continent of origin (Europe and others), study design (prospective and retrospective), and methods of imaging analyses (visual and semiquantitative evaluation), and the results were stable (**Supplementary Tables S16**–**S21**).

### 3.3 Diagnostic Value of MRI on Per-Patient Analysis

#### 3.3.1 Magnetic Field Strength of MRI

**Figure 3:**
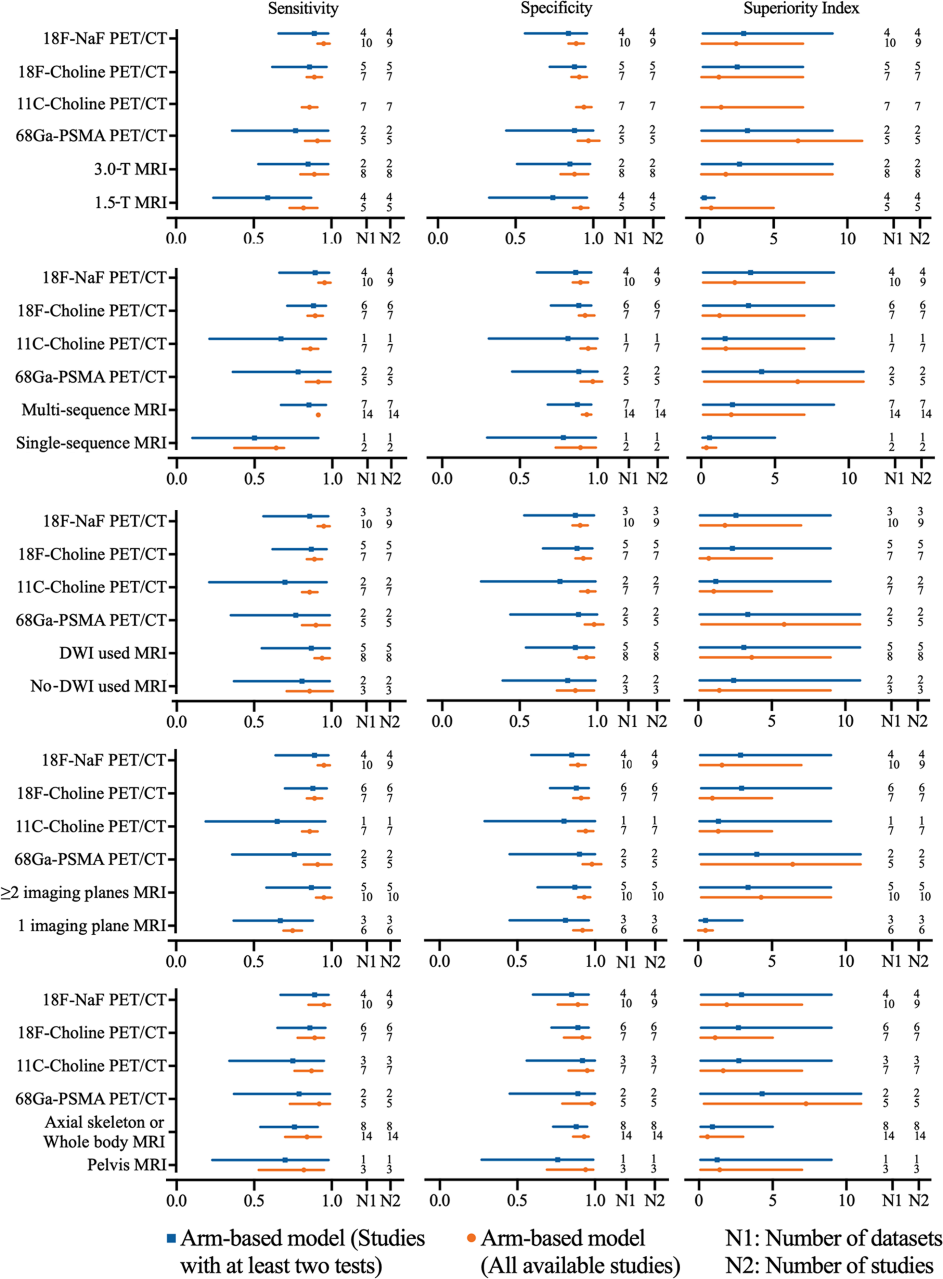
Network meta-analysis results including sensitivity, specificity, and superiority index values of PET/CT with four commonly used tracers and MRI with numerous characteristics for the detection of bone metastasis. Sensitivity, specificity, and superiority index are reported as mean (range) unless otherwise indicated. PET/CT, positron emission tomography/computed tomography; NaF, sodium fluoride; PSMA, prostate membrane antigen; DWI, diffusion-weighted imaging.

The pooled sensitivity and specificity of 1.5-T MRI were 0.82 (95% CI, 0.73–0.91) and 0.92 (95% CI, 0.87–0.97), respectively, while the pooled sensitivity and specificity of 3.0-T MRI were 0.89 (95% CI, 0.80–0.98) and 0.88 (95% CI, 0.79–0.97). Network meta-analysis demonstrated that 68Ga-PSMA PET/CT had the highest diagnostic value with the highest superiority index, followed closely by 18F-NaF PET/CT, 3.0-T MRI [1.76, (95% CI, 0.09–9.00)], 11C-choline PET/CT, 18F-choline PET/CT, and 1.5-T MRI [0.77, (95% CI, 0.09–5.00)] (**Supplementary Table S22** and **Figure 3**).

#### 3.3.2 Sequence of MRI

The pooled sensitivity and specificity of multisequence MRI were 0.91 (95% CI, 0.87–0.95) and 0.93 (95% CI, 0.90–0.96), while the pooled sensitivity and specificity of single-sequence MRI were 0.64 (95% CI, 0.48–0.80) and 0.89 (95% CI, 0.76–1.02), respectively. Network meta-analysis demonstrated that 68Ga-PSMA PET/CT had the highest diagnostic value with the highest superiority index, followed closely by 18F-NaF PET/CT, multisequence MRI [2.04, (95% CI, 0.14–7.00)], 11C-choline PET/CT, 18F-choline PET/CT, and single-sequence MRI [0.36, (95% CI, 0.09–1.02)] (**Supplementary Table S23** and **Figure 3**).

#### 3.3.3 Whether DWI Was Used

The pooled sensitivity and specificity of DWI MRI were 0.94 (95% CI, 0.89–0.99) and 0.93 (95% CI 0.88–0.98), while the pooled sensitivity and specificity of no-DWI MRI were 0.86 (95% CI, 0.71–1.01) and 0.86 (95% CI, 0.74–0.98), respectively. Network meta-analysis demonstrated that 68Ga-PSMA PET/CT had the highest diagnostic value with the highest superiority index, followed closely by DWI MRI [3.63, (95% CI 0.14–9.00)], 18F-NaF PET/CT, no-DWI MRI [1.44, (95% CI, 0.09–9.00)], 11C-choline PET/CT, and 18F-choline PET/CT (**Supplementary Table S24** and **Figure 3**).

#### 3.3.4 Imaging Plane of MRI

The pooled sensitivity and specificity of ≥2 imaging planes MRI were 0.95 (95% CI, 0.90–1.00) and 0.93 (95% CI, 0.89–0.97), while the pooled sensitivity and specificity of 1 imaging plane MRI were 0.75 (95% CI, 0.69–0.81) and 0.92 (95% CI, 0.86–0.98), respectively. Network meta-analysis revealed that 68Ga-PSMA PET/CT had the highest diagnostic value with the highest superiority index, followed closely by two or more imaging planes MRI [4.27, (95% CI, 0.20–9.00)], 18F-NaF PET/CT, 11C-choline PET/CT, 18F-choline PET/CT, and one imaging plane MRI [0.25, (95% CI, 0.09–1.00)] (**Supplemental Table S25** and **Figure 3**).

#### 3.3.5 MRI Coverage

The pooled results demonstrated that axial skeleton or whole-body skeleton MRI had similar sensitivity of 0.84 *vs.* 0.82 and specificity of 0.93 *vs.* 0.94, compared with pelvis MRI. Network meta-analysis demonstrated that 68Ga-PSMA PET/CT had the highest diagnostic value with the highest superiority index, followed closely by 18F-NaF PET/CT, 11C-choline PET/CT, pelvis skeleton MRI, 18F-choline PET/CT, and axial skeleton or whole-body skeleton MRI (**Supplementary Table S26** and **Figure 3**).

#### 3.3.6 Sensitivity Analysis

In five subgroup analyses above, we performed sensitivity analysis by deleting studies involving only one test for detecting bone metastasis, which show the similar results (**Supplementary Tables S27**–**S31**).

### 3.4 Diagnostic Value of High-Quality MRI *vs.* PET/CT on Per-Patient Analysis

**Figure 4:**
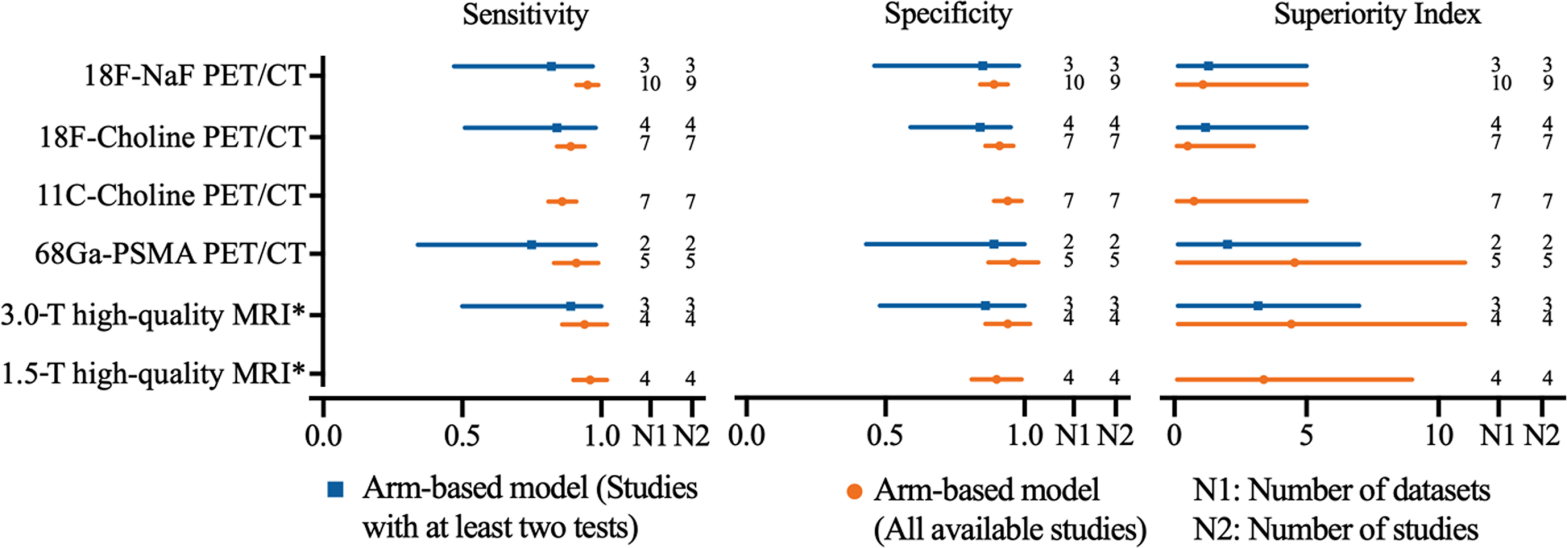
Network meta-analysis results including sensitivity, specificity, and superiority index values of PET/CT with four commonly used tracers and high-quality MRI for the detection of bone metastasis in prostate cancer patients. Sensitivity, specificity, and superiority index are reported as mean (range) unless otherwise indicated. PET/CT, positron emission tomography/computed tomography; NaF, sodium fluoride; PSMA, prostate membrane antigen; DWI diffusion-weighted imaging; *high-quality MRI was referred to the MRI equipped with multi-sequence, DWI used, and ≥2 imaging planes.

In order to achieve the highest accuracy of MRI, we define a high-quality MRI which is referred to the MRI equipped with multisequence, DWI used, and ≥2 imaging planes. High-quality MRI (1.5-T) has a sensitivity of 0.96 (95% CI, 0.90–1.02) and a specificity of 0.90 (95% CI, 0.81–0.99), while 3.0-T high-quality MRI has a sensitivity of 0.94 (95% CI, 0.86–1.02) and a specificity of 0.94 (95% CI, 0.86–1.02), respectively. Network meta-analysis demonstrates that 68Ga-PSMA PET/CT has the highest diagnostic value with the highest superiority index [4.56, (95% CI, 0.11–11.00)], followed closely by 3.0-T high-quality MRI [4.43, (95% CI, 0.14–11.00)], 1.5-T high-quality MRI [3.38, (95% CI, 0.11–9.00)], 18F-NaF PET/CT, 11C-choline PET/CT, and 18F-choline PET/CT (**Table 3**). The sensitivity analyses were performed by deleting studies involving only one test for detecting bone metastasis, and the results were stable (**Supplemental Table S32** and **Figure 4**).

**Table 3:** PET/CT with different tracers and high-quality MRI to detect bone metastasis in PCa.

Test	Absolute Sensitivity	Absolute Specificity	Diagnostic OR[Rank]	Superiority Index [Rank]	Relative Sensitivity	Relative Specificity	Datasets, n	Studies, n
18F-NaF PET/CT	0.95 (0.91–0.99)	0.89 (0.84–0.94)	256.89 (34.08–796.33) [4]	1.08 (0.11–5.00) [4]	1.00 (1.00–1.00)	1.00 (1.00–1.00)	10	9
18F-Choline PET/CT	0.89 (0.84–0.94)	0.91 (0.86–0.96)	121.19 (22.43–355.84) [6]	0.51 (0.09–3.00) [6]	0.94 (0.87–1.01)	1.03 (0.95–1.11)	7	7
11C-Choline PET/CT	0.86 (0.81–0.91)	0.94 (0.89–0.99)	212.63 (20.64–791.67) [5]	0.75 (0.09–5.00) [5]	0.91 (0.84–0.98)	1.07 (0.98–1.16)	7	7
68Ga-PSMA PET/CT	0.91 (0.83–0.99)	0.96 (0.87–1.05)	4,633,299.15 (14.51–4,438,033.15) [1]	4.56 (0.11–11.00) [1]	0.96 (0.86–1.06)	1.09 (0.96–1.22)	5	5
3.0-T high-quality MRI	0.94 (0.86–1.02)	0.94 (0.86–1.02)	6033.33 (27.09–39,719.47) [2]	4.43 (0.14–11.00) [2]	0.99 (0.89–1.09)	1.07 (0.96–1.18)	4	4
1.5-T high-quality MRI	0.96 (0.90–1.02)	0.90 (0.81–0.99)	2,056.83 (23.96–11,589.08) [3]	3.38 (0.11–9.00) [3]	1.02 (0.93–1.11)	1.02 (0.90–1.14)	4	4

Data are reported as mean (range) unless otherwise indicated.

PET/CT, positron emission tomography/computed tomography; MRI, magnetic resonance imaging; PCa, Prostate cancer; NaF, Sodium fluoride; PSMA, Prostate membrane antigen; T, Tesla; CI credible interval; OR, odds ratio.

High-quality MRI was referred to the MRI equipped with multisequence, DWI used, and ≥2 imaging planes.

### 3.5 Diagnostic Value of PET/CT *vs.* MRI on Per-Lesion Analysis

Regarding the tracers of the included studies, three studies used 18F-NaF, four selected 18F-choline, two applied 11C-choline, and one selected 68Ga-PSMA PET/CT. Only five studies reported MRI data. The pooled results demonstrated that 11C-choline PET/CT had the highest sensitivity of 0.84 (95% CI, 0.70–0.98), and 18F-choline PET/CT has the highest specificity of 0.91 (95% CI, 0.83–0.99). Network meta-analysis revealed that 11C-choline PET/CT had the highest diagnostic value with the highest superiority index, followed closely by 68Ga-PSMA PET/CT, 18F-choline PET/CT, MRI, and 18F-NaF PET/CT (**Supplementary eTable 33**). Subgroup analysis based on the analysis of high-quality MRI (only 1.5-T, insufficient data for 3.0-T high-quality MRI) showed similar results (**Supplementary Table S34**).

## 4 Discussion

“In the current meta-analysis, we compared the diagnostic accuracy of MRI and PET/CT for the detection of bone metastasis in PCa. On patient-based level, network meta-analysis reveals that for numerous tracers, 68Ga-PSMA PET/CT has the highest superiority index, followed by 18F-NaF, 11C-choline, 18F-choline, 18F-FDG, and 18F-fluciclovine PET/CT; for the parameters of MRI, higher magnetic field strength, multisequence, more imaging planes, and MRI with DWI will increase the diagnostic value for bone metastasis in prostate cancer patients. Where available, 3.0-T high-quality MRI approaches 68Ga-PSMA PET/CT was performed in the detection of bone metastasis (sensitivity, 0.94 *vs.* 0.91; specificity, 0.94 *vs.* 0.96; superiority index, 4.43 *vs.* 4.56).

Given this hybrid method, as PET provides metabolic information and morphological imaging techniques offer anatomical data (27, 28, 87, 88), a more accurate delineation of bone metastases is allowed. As well-known, the radioactive tracer is one of the most important cores of nuclear medicine imaging. Antoch et al. (28) demonstrated that the selection of the appropriate radioligands could increase accuracy when detecting micrometastases. Hence, we conducted the analysis based on the several popular tracers and found that 68Ga-PSMA PET/CT possessed the highest diagnostic value [superiority index, 7.30 (95% CI, 0.60–11.00)]. Pyka et al. (89) performed a retrospective study and showed a higher sensitivity and specificity of 68Ga-PSMA PET/CT (100% and 100%) when compared to BS. Uslu-Besli et al. (86) conducted a cohort study of 28 patients and demonstrated that 68Ga-PSMA PET/CT changed management of seven patients by confirming the presence of bone metastasis, which was overlooked by BS in three patients and by excluding the false negative lesions on BS in four patients. Through binding to PSMA, a transmembrane protein expressed predominantly in prostate cells and especially in prostate cancer cells, 68Ga-PSMA leads to the internalization and accumulation in primary and metastatic cancer lesions (90), which is proven to have the higher yield of positive scans than obtained with other tracers at a low PSA level (91, 92). However, the half-life and yield of 68Ga significantly limited the ability 68Ga-PSMA to meet the demand for imaging in PCa. To this regard, 18F-PSMA was considered as the ideal PET/CT tracer (93), but we were not able to analyze it because of the insufficient study.

Although the recommendation of the use of 18F-NaF PET/CT in PCa was refrained in guidelines, it is routinely used worldwide (94, 95). Our results showed that 18F-NaF PET/CT had the highest sensitivity (0.95) but relative low specificity (0.88). The uptake of 18F-NaF, a bone-specific imaging radiotracer, is correlated to the blood flow and, especially, to the activity of local osteoblasts (30, 96–98), and the study by the National Oncologic PET Registry (NOPR) showed that the intended managements in approximately 44–53% of prostate cancer patients had been significantly impacted by the application of 18F-Na PET/CT (99, 100). However, 18F-NaF has the same shortcoming as the other bone-seeking agents, such as 99m-technetium used in BS, leading to confusion between benign lesion and metastases, and which creates more false positives and causes lower specificity (78, 101). Choline is an essential component of the phospholipids, whose increase is associated with the high proliferation of prostate cancer cells, and both 11C-Choline and 18F-choline have been investigated in particular for the detection of relapse and metastasis of PCa (102–104). Our results demonstrated that the urinary excretion of 18F-choline was slightly higher than 11C-Choline (superiority index, 1.92 *vs.* 1.71), which may affect the interpretation of findings in the pelvis and cause lower accuracy (105, 106). Additionally, our study validated that 11C-Choline PET/CT processed the highest sensitivity–specificity and superiority index on the per-lesion basis (preformed in a situation of the lack the data of 68GA-PMSA PET/CT). The commonly used tracer 18F-FDG appears to be less useful in PCa because of the low avidity of most prostate cancer cells and urinary activity (107). Osseous metastases in PCa are typically osteoblastic (7, 108), while 18F-FDG are more sensitive in osteolytic lesions than in osteogenic lesions (72).

According to different factors (magnetic field strength, coverage, sequence used, the participation of DWI, and the number of imaging planes), MRI was grouped and directly compared to PET/CT using four commonly used radioligands (68Ga-PSMA, 18F-NaF, 11C-choline, and 18F-choline). 68Ga-PSMA PET/CT was still predominating, which was followed by the MRI equipped with better options. It is possible for MRI to detect metastasis lesion at an early stage owing to the high soft-tissue resolution (109–111). Meanwhile, numerous clinical advantages of 3.0-T MRI over 1.5-T have been demonstrated (112), which was also confirmed in our analysis (sensitivity, 0.89 *vs.* 0.82; specificity, 0.88 *vs.* 0.92; superiority index, 1.76 *vs.* 0.77). However, Woo et al. (22) reported that there existed no significant heterogeneity among different magnetic field strength. Recently, DWI is of increasing interest for the detection of primary or metastatic cancers (113, 114), benefiting from the ability to differentiate malignant from benign prostatic tissues according to different water diffusivity (68). Our analysis, according to whether DWI was involved, indicated the usefulness of DWI in the evaluation of bone metastases in PCa (sensitivity, 0.94 *vs.* 0.86; specificity, 0.93 *vs.* 0.86; superiority index, 3.63 *vs.* 1.44). A previous meta-analysis reported similar results that of improved diagnostic performance for identifying tumor foci in PCa due to the useful complement from DWI (115). Barchetti et al. (68) also revealed that conventional imaging, including T1-weighted (T1W), T1-weighted images (T2W), and short tau inversion recovery (STIR) sequence, may improve the specificity of DWI in detecting bone metastases. Regarding the coverage of MRI, whether a dedicated axial skeleton or whole-body MRI was utilized or only covered the pelvis, the results showed a similar performance (sensitivity, 0.44 *vs.* 0.82; specificity, 0.94 *vs.* 0.93). Although it is necessary to assess the extent of extra-prostatic extension, which is an independent prognostic factor (11), the possibility of distant bone metastasis without pelvic or lumbar spinal involvement is negligible (44, 116). The motion-related signal intensity also decreases, leading to the failure in depicting lesions in ribs, sternum, and scapula (117, 118), and the increased cost and acquisition time remain obstacles to the use of WB-MRI. However, the application of wider coverage enables the detection of extra-skeletal involvement, including lymph nodes (119, 120), allows their monitoring under therapy, and helps to assess the efficacy of many new drugs in advanced PCa (121–123). Additionally, the use of more imaging planes and sequences to determine bone metastases can achieve better diagnostic accuracy, attributed to the acquisition of more information. These analyses proved that the optimization of MRI parameters could significantly improve the diagnostic ability of bone metastasis in PCa.

The highlight of the current meta-analysis was to directly compare the high-quality MRI (multisequence, DWI involved, more imaging planes) with PET/CT using four commonly used tracers. The results showed that although the 3.0-T high-quality MRI did not surpass the 68Ga-PSMA PET/CT at the patient-based level, the diagnostic ability was very close (sensitivity, 0.94 *vs.* 0.91; specificity, 0.94 *vs.* 0.96; superiority index, 4.43 *vs.* 4.56). In recent decades, MRI and PET/CT compete for the single-step whole-body technique for assessing metastases and imaging of response to treatment in solid cancers. Lecouvet et al. (44) demonstrated that MRI was a highly sensitive and specific one-step modality to diagnose bone metastases in patients with high-risk PCa and leads to changes in treatment strategy in 22% of patients. Echmann et al. (43) proved the similarly high accuracy of 11C-Choline PET/CT and 1.5-T multiparametric WB-MRI, while Conde-Moreno et al. (124) found a significantly lower ability of DWI WB-MRI for detecting bone metastasis in recurrent PCa. Although previous studies have yielded inconsistent conclusions, our results demonstrated that 3.0-T multiparametric WB-MRI has a comparable high sensitivity, specificity, and superiority index at the patient-based level, following the 68Ga-PSMA PET/CT. Nevertheless, multiparametric WB-MRI seems to fulfill the requirements of no ionizing radiation and no intravenous injection of isotopes or any contrast medium, and it is also adept at depicting all metastatic bone lesions. T1W imaging can describe the infiltration of the bone marrow, T2W imaging has also been the mainstay of MRI due to its high tissue contrast resolution, while DWI can help discover the areas of increased cellularity and highly vascularized structures (71, 125, 126). Additionally, the efforts for standardization of prostate MRI acquisition and reporting (127, 128), including MR Prostate Imaging Reporting and Data System (PI-RADS) (129) and the Prostate Diagnostic Imaging Consensus Meeting (PREDICT) (130), have further contributed to the use and emphasized the importance of interpreting MRI in the context of clinical features.

### 4.1 Strengths and Limitations

The arm-based model is more appealing than traditional meta-analysis and the contrast-based model since the former not only permits more straightforward interpretation of the parameters, making use of all available data and yielding shorter credible intervals but also provides more natural variance–covariance matrix structures. We adopted this model, which makes our results more convincing.

We adopted numerous statistical indicators including absolute sensitivity and specificity, relative sensitivity and specificity, diagnostic odds ratio (DOR), and superiority index to compare the diagnostic value of PET/CT and MRI with different parameter systematically. It is still a challenge to rank competing diagnostic tests especially when a test does not outperform the others on both sensitivity and specificity. DOR is commonly used in traditional meta-analysis, but it cannot distinguish between tests with high sensitivity but low specificity or vice versa. Deutsch et al. (131) introduced a superiority index to quantify the superiority of a diagnostic test. The superiority index is designed to consider the joint performance of the assessment measures. Corresponding weight is given to diagnostic tests based on their performance.

Our network meta-analysis included a total of 45 studies involving 2,843 patients and 4,263 lesions, which, to our knowledge, is the largest among similar studies. While the data in our study can be used to demonstrate numerical superiority of one imaging modality in terms of sensitivity, specificity, etc., they are even richer in that these variables can be compared across all nine imaging modalities simultaneously to assess their relative accuracy. Using this large amount of diagnostic data, we compared these imaging modalities at patient- and lesion-based level. Furthermore, a series of subgroup analyses were conducted to explore the potential influencing factors. Other subgroup analysis suggested a similar direction and magnitude of effect for studies investigating diagnostic value. We also performed sensitivity analyses by removing studies involving only one diagnostic test to confirm the stability of our results. Our results provide a comprehensive overview of the existing evidence on the imaging diagnosis of bone metastasis and have implications for clinicians, researchers, radiologists, and guideline committees. While many oncologists already consider PET/CT as the preferred method for detecting bone metastasis, however, this study provide formal quantification of the relative value of MRI.

This study is not without weakness. The first limitation was the lack of a well-accepted reference standard; all included studies used best value comparator (BVC) or predominantly BVC as the reference standard, which is according to a combination of clinical, laboratory, imaging, and follow-up studies (22). Although it is a more accurate reference standard to obtain pathological results by biopsy or surgery when determining bone metastasis, it is neither feasible nor ethical to conduct such further examinations solely. Second, patients were categorized at diagnosis to low or high risk based on clinical characteristics, such as PSA, Gleason score at biopsy, and clinical stage. It should be taken into account the potential value of the respective modalities according to the cancer grade and stage. For example, several studies reported that PSA values is strictly correlated with 11C-choline PET/CT sensitivity (102, 132). Hence, the specific imaging strategies should be adopted for PCa with different risk (133–135). Unfortunately, due to insufficient raw data, we are unable to do more subgroup analysis. Additionally, the same limitation made a comparison based on different metastatic sites difficult to accomplish. It is challenging for both PET/CT and MRI to diagnose rib metastases due to thoracic respiratory movements (103, 136–138), and further research on the diagnostic efficacy of different imaging modalities on thoracic sites will be interesting. Third, several other imaging modalities, such as BS, X-ray, BS/TXR, CT, PET, SPECT/CT, and especially, PET/MRI, a potentially disruptive technology synergizing PET and MRI, are also commonly used to assess bone metastasis. However, the purpose of this study was only to compare PET/CT and MRI in detecting bone metastasis in PCa; hence, caution is needed in applying our results to routine clinical practice. Fourth, studies assessing cost effectiveness should be performed to assist in making the clinical decision. In our study, insufficient data prevented us from assessing the economic effectiveness and social benefits systematically.

## 5 Conclusion

This systematic review and network meta-analysis of diagnostic tests, which included 45 studies involving 2,843 patients and 4,263 lesions, indicates that 68Ga-PSMA PET/CT is recommended for the diagnosis of bone metastasis in prostate cancer patients. Where available, 3.0-T high-quality MRI approaches 68Ga-PSMA PET/CT should be performed the detection of bone metastasis.

## Data Availability Statement

The original contributions presented in the study are included in the article/**Supplementary Material**. Further inquiries can be directed to the corresponding author.

## Author Contributions

FL, JD, and FX conceived and designed the project. FL, JD, and FX supervised the project. FL, JD, and FX performed the review and approval of the manuscript. FL, JD, DZ, YS, CY, RW, GW, JT, QY, BW, LL, JM, and FX contributed to the design of the study, writing the protocol, screening trials, data extraction, analysis and interpretation, and writing and final approval of the report. FL, JD, and FX generated the tables and figures. FL, JD, and FX assessed the quality of included trials. FL, JD, and FX performed the literature search. FL, JD, DZ, YS, CY, RW, GW, JT, QY, BW, LL, JM, and FX drafted the manuscript. FL, JD, DZ, YS, CY, RW, GW, JT, QY, BW, LL, JM, and FX participated in revising the manuscript before submission. FL, JD, DZ, YS, CY, RW, GW, JT, QY, BW, LL, JM, and FX participated in the formal revision, including data processing, statistical analysis, generating figures and tables, and text modification. All authors had full access to the data in the study and can take responsibility for the integrity of the data and the accuracy of the data analysis. FX is the guarantor. The corresponding author attests that all listed authors meet authorship criteria and that no others meeting the criteria have been omitted. All authors contributed to the article and approved the submitted version.

## Funding

This study was supported by China Scholarship Council, Nos. 201808080126 and 201706920036; the National Natural Science Foundation of China, No. 81301556; and the Key R&D program in Shandong Province, No. 2016GSF201214. The sponsors or funders had no involvements in any parts of this study.

## Conflict of Interest

The authors declare that the research was conducted in the absence of any commercial or financial relationships that could be construed as a potential conflict of interest.

## Publisher’s Note

All claims expressed in this article are solely those of the authors and do not necessarily represent those of their affiliated organizations, or those of the publisher, the editors and the reviewers. Any product that may be evaluated in this article, or claim that may be made by its manufacturer, is not guaranteed or endorsed by the publisher.

## Supplementary Material

Supplementary Table 1 Definitions of outcomes.

Supplementary Table 2 Search strategy.

Supplementary Table 3 The extraction process of diagnostic data.

Supplementary Table 4 The detail methods of QUADAS-2 score.

Supplementary Table 5 Implementation methods and process using arm-based model for network meta-analysis of diagnostic test accuracy data.

Supplementary Table 6 Main characteristics of MRI in the included studies.

Supplementary Table 7 Main characteristics of PET/CT in the included studies.

Supplementary Table 8 Diagnostic data of the included studies on patient-based level.

Supplementary Table 9 Diagnostic data of the included studies on lesion-based level.

Supplementary Table 10 The results of quality assessment of the included studies.

Supplementary Table 11 PET/CT with different tracers for detecting bone metastasis in patients with PCa by deleting studies with only one diagnostic test.

Supplementary Table 12 PET/CT with different tracers for detecting bone metastasis in patients with PCa by deleting studies with a QUADAS-2 score of 7.

Supplementary Table 13 PET/CT with different tracers for detecting bone metastasis in patients with PCa by deleting study with the maximum sample size.

Supplementary Table 14 PET/CT with different tracers for detecting bone metastasis in patients with PCa by deleting study with the minimum sample size.

Supplementary Table 15 PET/CT with different tracers for detecting bone metastasis in patients with PCa by detecting studies published before 2010.

Supplementary Table 16 PET/CT with different tracers for detecting bone metastasis in patients with PCa based on clinical settings of cancers.

Supplementary Table 17 PET/CT with different trances for detecting bone metastasis in patients with PCa based on numbers of patients.

Supplementary Table 18 PET/CT with different tracers for detecting bone metastasis in patients with PCa based on ages of patients.

Supplementary Table 19 PET/CT with different tracers for detecting bone metastasis in patients with PCa based on continents of studies.

Supplementary Table 20 PET/CT with different tracers for detecting bone metastasis in patients with PCa based on study design.

Supplementary Table 21 PET/CT with different tracers for detecting bone metastasis in patients with PCa based on evaluation methods of imaging analyses.

Supplementary Table 22 PET/CT with different tracers and MRI with different field strength for detecting bone metastasis in patients with PCa.

Supplementary Table 23 PET/CT with different tracers and MRI with different numbers of sequences for detecting bone metastasis in patients with PCa.

Supplementary Table 24 PET/CT with different tracers and MRI with/without DWI for detecting bone metastasis in patients with PCa.

Supplementary Table 25 PET/CT with different tracers and MRI with different numbers of imaging planes for detecting bone metastasis in patients with PCa.

Supplementary Table 26 PET/CT with different tracers and MRI with different coverage for detecting bone metastasis in patients with PCa.

Supplementary Table 27 PET/CT with different tracers and MRI with different field strength for detecting bone metastasis in patients with PCa by deleting studies with only one diagnostic test.

Supplementary Table 28 PET/CT with different tracers and MRI with different numbers of sequences for detecting bone metastasis in patients with PCa by deleting studies with only one diagnostic test.

Supplementary Table 29 PET/CT with different tracers and MRI with/without DWI for detecting bone metastasis in patients with PCa by deleting studies with only one diagnostic test.

Supplementary Table 30 PET/CT with different tracers and MRI with different numbers of imaging planes for detecting bone metastasis in patients with PCa by deleting studies with only one diagnostic test.

Supplementary Table 31 PET/CT with different tracers and MRI with different coverage for detecting bone metastasis in patients with PCa by deleting studies with only one diagnostic test.

Supplementary Table 32 PET/CT with different tracers and 3.0-T high-quality MRI for detecting bone metastasis in patients with PCa by deleting studies with only one diagnostic test.

Supplementary Table 33 PET/CT with different tracers and MRI for detecting bone metastasis in patients with PCa on lesion-based level.

Supplementary Table 34 PET/CT with different tracers and 1.5-T high-quality MRI for detecting bone metastasis in patients with PCa on lesion-based level.
