# Supplementary material for: Comparison of PET/CT and MRI in the Diagnosis of Bone Metastasis in Prostate Cancer Patients: A Network Analysis of Diagnostic Studies
Source: Front Oncol. 2021 Oct 4;11:736654. doi: 10.3389/fonc.2021.736654 (PMC8522477; doi:10.3389/fonc.2021.736654)
Supplement: Supplementary Table 1 — Definitions of outcomes. [file DataSheet_1.docx]

**Supplemental e-material**

eTable 1. Definitions of outcomes

eTable 2. Search strategy

eTable 3. The extraction process of diagnostic data

eTable 4. The detail methods of QUADAS-2 score

eTable 5. Implementation methods and process using arm-based model for network meta-analysis of diagnostic test accuracy data

eTable 6. Main characteristics of MRI in the included studies

eTable 7. Main characteristics of PET/CT in the included studies

eTable 8. Diagnostic data of the included studies on patient-based level

eTable 9. Diagnostic data of the included studies on lesion-based level

eTable 10. The results of quality assessment of the included studies

eTable 11. PET/CT with different tracers for detecting bone metastasis in patients with PCa by deleting studies with only one diagnostic test

eTable 12. PET/CT with different tracers for detecting bone metastasis in patients with PCa by deleting studies with a QUADAS-2 score of 7

eTable 13. PET/CT with different tracers for detecting bone metastasis in patients with PCa by deleting study with the maximum sample size

eTable 14. PET/CT with different tracers for detecting bone metastasis in patients with PCa by deleting study with the minimum sample size

eTable 15. PET/CT with different tracers for detecting bone metastasis in patients with PCa by detecting studies published before 2010

eTable 16. PET/CT with different tracers for detecting bone metastasis in patients with PCa based on clinical settings of cancers

eTable 17. PET/CT with different trances for detecting bone metastasis in patients with PCa based on numbers of patients

eTable 18. PET/CT with different tracers for detecting bone metastasis in patients with PCa based on ages of patients

eTable 19. PET/CT with different tracers for detecting bone metastasis in patients with PCa based on continents of studies

eTable 20. PET/CT with different tracers for detecting bone metastasis in patients with PCa based on study design

eTable 21. PET/CT with different tracers for detecting bone metastasis in patients with PCa based on evaluation methods of imaging analyses

eTable 22. PET/CT with different tracers and MRI with different field strength for detecting bone metastasis in patients with PCa

eTable 23. PET/CT with different tracers and MRI with different numbers of sequences for detecting bone metastasis in patients with PCa

eTable 24. PET/CT with different tracers and MRI with/without DWI for detecting bone metastasis in patients with PCa

eTable 25. PET/CT with different tracers and MRI with different numbers of imaging planes for detecting bone metastasis in patients with PCa

eTable 26. PET/CT with different tracers and MRI with different coverage for detecting bone metastasis in patients with PCa

eTable 27. PET/CT with different tracers and MRI with different field strength for detecting bone metastasis in patients with PCa by deleting studies with only one diagnostic test

eTable 28. PET/CT with different tracers and MRI with different numbers of sequences for detecting bone metastasis in patients with PCa by deleting studies with only one diagnostic test

eTable 29. PET/CT with different tracers and MRI with/without DWI for detecting bone metastasis in patients with PCa by deleting studies with only one diagnostic test

eTable 30. PET/CT with different tracers and MRI with different numbers of imaging planes for detecting bone metastasis in patients with PCa by deleting studies with only one diagnostic test

eTable 31. PET/CT with different tracers and MRI with different coverage for detecting bone metastasis in patients with PCa by deleting studies with only one diagnostic test

eTable 32. PET/CT with different tracers and 3.0-T high-quality MRI for detecting bone metastasis in patients with PCa by deleting studies with only one diagnostic test

eTable 33. PET/CT with different tracers and MRI for detecting bone metastasis in patients with PCa on lesion-based level

eTable 34. PET/CT with different tracers and 1.5-T high-quality MRI for detecting bone metastasis in patients with PCa on lesion-based level

| **eTable 1. Definitions of outcomes** | |
| --- | --- |
| **Outcomes** | **Definitions** |
| Absolute Sensitivity | Sensitivity (also called the true positive rate, the recall, or probability of detection in some fields) measures the proportion of actual positives that are correctly identified as such (e.g., the percentage of sick people who are correctly identified as having the condition). |
| Absolute Specificity | Specificity relates to the test's ability to correctly reject healthy patients without a condition. Consider the example of a medical test for diagnosing a disease. Specificity of a test is the proportion of healthy patients known not to have the disease, who will test negative for it. |
| Relative Sensitivity | Relative Sensitivity is a measure of a test compared to another or differences in accuracy between tests, which are easily interpretable and often used in clinical epidemiology. |
| Relative Specificity | Relative Specificity is a measure of a test compared to another or differences in accuracy between tests, which are easily interpretable and often used in clinical epidemiology. |
| Diagnostic odds ratio (DOR) | Diagnostic odds ratio is a measure of the effectiveness of a diagnostic test. It is defined as the ratio of the odds of the test being positive if the subject has a disease relative to the odds of the test being positive if the subject does not have the disease. |
| Superiority Index (S) | The superiority of a diagnostic test could be quantified using a superiority index expressed as S*_k_*= (2a*_k_*+c*_k_*)/(2b*_k_*+c*_k_*) where a*_k_* is the number of tests to which test *k* is superior (higher sensitivity and specificity), b*_k_* is the number of tests to which test k is inferior (lower sensitivity and specificity) and c*_k_* the number of tests with equal performance as test *k* (equal sensitivity and specificity). S ranges from 0 to 1 with S tending to 1 and S tending to 0 as the number of tests to which test k is superior and inferior increases, respectively, and S tending to 1 the more the tests are equal. Since the number of tests not comparable to test *k* does not enter into the calculation of S, the index for different tests may be based on different sets of tests. |

| **eTable 2. Search strategy** | | |
| --- | --- | --- |
| **MEDLINE (R)** | | |
| 1 | (prostate cancer?) AND (bone metastasis? OR bone? OR distant metastasis? OR recurrence?) AND (diagnostic? OR diagnosis? OR detect?) | 15552 |
| 2 | Humans.pt. | 14772 |
| **Embase** | | |
| 1 | ('prostate cancer'/exp OR 'prostate cancer' OR (('prostate'/exp OR prostate) AND ('cancer'/exp OR cancer))) AND ('bone metastasis'/exp OR 'bone metastasis' OR 'metastasis'/exp OR metastasis OR 'bone'/exp OR bone OR 'distant metastasis'/exp OR 'distant metastasis' OR (distant AND ('metastasis'/exp OR metastasis)) OR 'recurrence'/exp OR recurrence) AND ('diagnostic'/exp OR diagnostic OR 'diagnosis'/exp OR diagnosis OR detect) | 41533 |
| 2 | [humans]/lim | 38067 |
| 3 | [clinical study]/lim | 24218 |
| 4 | [controlled clinical trial]/lim | 2035 |
| 5 | [randomized controlled trial]/lim | 835 |
| 6 | 4 or 5 | 2035 |
| **EBM Reviews - Cochrane Central Register of Controlled Trials** | | |
| 1 | (Prostate cancer?) AND (bone metastasis? OR bone? OR distant metastasis? OR recurrence?) AND (diagnostic? OR diagnosis? OR detect?).af. | 7719 |
| 2 | Trials | 7684 |

| **eTable 3. The extraction process of diagnostic data** | |
| --- | --- |
| **1. Reports presented original data** | If the original data was presented by the author in the articles, then the true positive (TP), the true negative (TN), the false positive (FP), and the false negative (FN) can be extracted directly.  Take the article of Venkitaraman et al.^1^ as an example. The Table 2 of their article presented predictive values of detecting spinal metastasis by radionuclide bone scan and magnetic resonance imaging (MRI) of the spine for individual patients compared with the ‘gold standard’. We got the results of 99 patients for detecting BM using MRI, which were TP: 12, FP: 2, TN: 2, and FN: 83. |
| **2. Reports presented information for each patient** | If the diagnostic results of imaging methods and the gold standard methods of each patient were listed separately, then the TP, TN, FP, and FN can be extracted after simple statistics.  Take the trial of Jadvar et al.^2^ as an example. The Table 1 of this article presented the diagnostic results of PET/CT in detecting bone metastases of each patient. After comparing to the gold standard, the authors provided PET/CT results for 37 patients respectively. And the results were TP: 3, FP: 0, TN: 11, and FN: 23. |
| **3. Reports presented sensitivity, specificity, accuracy, and the number of total patients** | If the sensitivity (Se), specificity (Sp), accuracy (Acc), and the number of total patient (N) were presented in the trial reports, the TP, TN, FP, and FN can be calculated directly:  $TP=\frac{Se*Sp-Se*Acc}{Sp-Se}*N$  $FP=\frac{[Acc-\frac{Se*\left( Sp-Acc \right)}{Sp-Se}]* \left( 1-Sp \right)}{\mathrm{Sp}}*N$  $FN=\frac{(1-Se)*(Sp-Acc)}{Sp-Se}*N$  $TN=[Acc -\frac{Se*\left( Sp-Acc \right)}{Sp-Se}]*N$  Take the article of Kitajima et al.^3^ as an example. We extracted the Se, Sp, Acc, and N of MRI from Table 4, and then we can calculated the data we need:  The Se, Sp, Acc, and N of MRI in diagnosis of BM were 0.875, 0.962, 0.947, and 95 respectively.  $TP=\frac{0.875*0.962-0.875*0.947}{0.962-0.875}*95=14$  $FP=\frac{[0.947-\frac{0.875*\left( 0.962-0.947 \right)}{0.962-0.875}]* \left( 1-0.962 \right)}{0.962}*95=3$  $FN=\frac{(1-0.875)*(0.962-0.947)}{0.962-0.875}*95=2$  $TN=\left[ 0.947 -\frac{0.875*\left( 0.962-0.947 \right)}{0.962-0.875} \right]*95=76$  So we got the results of 95 patients for detecting BM using PET/CT, which were TP: 14, FP: 3, TN: 2, and FN: 76. |
| **4. Reports presented sensitivity, specificity, positive/negative predictive value, and the number of total patients** | If the Se, Sp, positive/negative predictive value (PPV/NPV), and N were presented in the trial reports, the TP, TN, FP, and FN can be calculated directly:  $TP=\frac{PPV*Se*(1-Sp)}{Se+PPV-PPV*(Se+Sp)}*N$  $FP=\frac{Se*(1-PPV)*\left( 1-Sp \right)}{Se+PPV-PPV*(Se+Sp)}*N$  $FN=\frac{PPV*\left( 1-Sp \right)*(1-Se)}{Se+PPV-PPV*(Se+Sp)}*N$  $TN=\frac{Se*Sp*(1-Sp)}{Se+PPV-PPV*(Se+Sp)}*N$  OR  $TP=\frac{Sp*Se*(1-NPV)}{Sp+NPV-NPV*(Se+Sp)}*N$  $FP=\frac{NPV*(1-Se)*\left( 1-Sp \right)}{Sp+NPV-NPV*(Se+Sp)}*N$  $FN=\frac{Sp*\left( 1-NPV \right)*(1-Se)}{Sp+NPV-NPV*(Se+Sp)}*N$  $TN=\frac{NPV*Sp*(1-Se)}{Sp+NPV-NPV*(Se+Sp)}*N$  Take the article of Conde-Moreno et al.^4^ as an example. We extracted the Se, Sp, PPV, and N of PET/CT from Table 6, and then we can calculated the data we need:  The Se, Sp, PPV, and N of PET/CT in diagnosis of BM in patients with prostate cancer were 0.8800, 0.8846, 0.8800, and 35 respectively.  $TP=\frac{0.8800*0.8800*(1-0.8846)}{0.8800+0.8800-0.8800*(0.8800+0.8846)}*35=15$  $FP=\frac{0.8800*(1-0.8800)*\left( 1-0.8846 \right)}{0.8800+0.8800-0.8800*(0.8800+0.8846)}*35=2$  $FN=\frac{0.8800*\left( 1-0.8846 \right)*(1-0.8800)}{0.8800+0.8800-0.8800*(0.8800+0.8846)}*35=2$  $TN=\frac{0.8800*0.8846*(1-0.8800)}{0.8800+0.8800-0.8800*(0.8800+0.8846)}*35=16$  So the data of PET/CT in diagnosis of BM in patients with prostate cancer were TP: 15, FP: 2, TN: 2, and FN: 16. |

**References**

1. Venkitaraman R, Cook GJ, Dearnaley DP, et al. Does magnetic resonance imaging of the spine have a role in the staging of prostate cancer? *Clin Oncol (R Coll Radiol)* 2009;21(1):39-42. doi: 10.1016/j.clon.2008.09.006

2. Jadvar H, Desai B, Ji L, et al. Prospective evaluation of 18F-NaF and 18F-FDG PET/CT in detection of occult metastatic disease in biochemical recurrence of prostate cancer. *Clin Nucl Med* 2012;37(7):637-43. doi: 10.1097/RLU.0b013e318252d829 [published Online First: 2012/06/14]

3. Kitajima K, Murphy RC, Nathan MA, et al. Detection of recurrent prostate cancer after radical prostatectomy: comparison of 11C-choline PET/CT with pelvic multiparametric MR imaging with endorectal coil. *J Nucl Med* 2014;55(2):223-32. doi: 10.2967/jnumed.113.123018

4. Conde-Moreno AJ, Herrando-Parreno G, Muelas-Soria R, et al. Whole-body diffusion-weighted magnetic resonance imaging (WB-DW-MRI) vs choline-positron emission tomography-computed tomography (choline-PET/CT) for selecting treatments in recurrent prostate cancer. *Clin Transl Oncol* 2016;19(5):553-61. doi: 10.1007/s12094-016-1563-4

| **eTable 4. The detail methods of QUADAS-2 score** | |
| --- | --- |
| **DOMAIN 1: PATIENT SELECTION** | |
| Was a consecutive or random sample of patients enrolled? | 1. An ideal study should include consecutive or random inclusion of eligible patients with suspected disease to avoid potential bias. If the inclusion of the case in the literature was continuous and had a time category, then it was rated as “Yes”; 2. if nothing was introduced, it was rated as “No”; 3. if there was a time limit for inclusion, but there was no indication of continuity, it was rated as "Unclear." |
| Was a case-control design avoided? | 1. Among the included cases, at least one case in a certain group was diagnosed as uncertain, then it was rated as “Yes”; 2. if one group could be diagnosed directly and the other group was suspected, then it was rated as “No”; 3. if insufficient information was provided, it was judged as "Unclear". |
| Did the study avoid inappropriate exclusions? | 1. Studies with inappropriate exclusion may lead to overestimation of diagnostic accuracy, and studies involving known diseases and inconsistent controls can also exaggerate diagnostic accuracy; conversely, excluding easy-to-diagnose patients may lead to underestimation of diagnostic accuracy. Among the included cases, if the cases with uncertain diagnosis accounted for 20%-30% or more, then it was rated as “Yes”; 2. if they could be diagnosed clearly, which means they did not include cases with uncertain diagnosis, then it was rated as "no"; 3. if the cases with uncertain diagnosis was less than 20%, then it was rated as "Unclear". |
| **DOMAIN 2: INDEX TEST(S)** | |
| Were the index test results interpreted without knowledge of the results of the reference standard? | The blindness method should be followed in judging the results of tests to be evaluated, because the information of the reference standard may affect the interpretation of the tests. If the test was always implemented and explained before the results of the reference standard was known, the item can be rated as “Yes” and vice versa as “No”; if not, it was “Unclear”. |
| If a threshold was used, was it pre-specified? | Choosing a test threshold to optimize sensitivity and/or specificity may result in an improved diagnostic performance; in independent samples, using the same threshold for the patient may degrade diagnostic performance. If the threshold used was determined before the evaluation, then it was rated as “Yes”, and vice versa as “No”; if the information was insufficient, then it was rated as "Unclear". |
| **DOMAIN 3: REFERENCE STANDARD** | |
| Is the reference standard likely to correctly classify the target condition? | 1. If the reference standard can correctly distinguish the target disease or is already the best method available, it was judged as “Yes”; 2. if it cannot be accurately distinguished, it was “No”; 3. if the information was insufficient, then it was rated as "Unclear". |
| Were the reference standard results interpreted without knowledge of the results of the index test? | If the interpretation of the reference standard results were performed without knowing the results of the tests to be evaluated, then it was rated as “Yes”, and vice versa as “No”; if the information was insufficient, then it was rated as "Unclear". |
| **DOMAIN 4: FLOW AND TIMING** | |
| Was there an appropriate interval between index test(s) and reference standard? | 1. The time interval leading to high-risk bias varies with the condition of the disease, and the key to determining this problem depends on the target disease. For acute diseases, if the time interval was very short, it was rated as “Yes”, while for chronic diseases, it should be evaluated as “Yes” even if the detection interval was long; 2. if the time interval between the reference standard and the test to be evaluated was too long, and the situation of target disease may have changed, then it was evaluated as “No”; 3. if the information was insufficient, then it was rated as "Unclear". |
| Did all patients receive a reference standard? | If it can be clearly judged that all patients have used the reference standard to verify their disease state, then it was rated as “Yes”, and vice versa as “No”; if the information was insufficient, then it was rated as "Unclear". |
| Did patients receive the same reference standard? | If the reference standard used by all patient was the same one, then it was rated as “Yes”, and vice versa as “No”; if the information was insufficient, then it was rated as "Unclear". |
| Were all patients included in the analysis? | 1. If all cases were included in the study and evaluated, then it was rated as “Yes”; 2. if there was a case omission, then it was rated as “No” ; 3. if the information was insufficient, then it was rated as "Unclear". |

| **eTable 5. Implementation methods and process using arm-based model for network meta-analysis of diagnostic test accuracy data** | | |
| --- | --- | --- |
| **Software** | R (v3.4.3; Comprehensive R Archive Network) | package rstan (v2.17.3), package loo (v2.0.0), package plyr, package ggplot2, package StanHeaders, package Rcpp, package RcppEigen, package BH, package methods, package stats4, package inline, package gridExtra, package Matrix and package parallel. |
| **Model** | rstan 2.17.3 package | The models are fitted in the Bayesian framework using Stan, a probabilistic programming language which has implemented Hamilton Monte Carlo (MHC) and No-U-Turn sampler (NUTS)^1^ within R 3.4.3 using the rstan 2.17.3 package.^2^ |
|  | process | 1. We run three chains in parallel until there is convergence. Trace plots are used to visually check whether the distributions of the three simulated chains mix properly and are stationary. 2. For each parameter, convergence is assessed by examining the potential scale reduction factor ^R, the effective number of independent simulation draws (n^eff^), and the MCMC error. 3. It is common practice to run simulations until ^R is no greater than 1.1 for all the parameters. Since Markov chain simulations tend to be autocorrelated, n^eff^ is usually smaller compared to the total number of draws. 4. To reduce autocorrelation and consequently increase n^eff^, it is necessary to do thinning by keeping every nth (e.g. every 10th, 20th, 30th. . .) draw and discarding the rest of the samples. Besides, thinning saves memory especially when the total number of iterations is large. |

**References**

1 MD H, A G. The no-u-turn sampler: Adaptively setting path lengths in hamiltonian monte carlo. *J Mach Learn Res* 2014;15:30.

2 RC T. A Language and environment for statistical computing. R Foundation for Statistical Computing. Vienna, Austria. *http://wwwR-projectorg/ (2019, accessed 31 January 2019).*

| **eTable 6. Main characteristics of MRI in the included studies** | | | | | | | | | | | | | | | | |
| --- | --- | --- | --- | --- | --- | --- | --- | --- | --- | --- | --- | --- | --- | --- | --- | --- |
| Study, year | Magnet strength  (T) | Vendor | Machine | Coverage | Sequence used | No. of  Imaging  planes | Minimum  ST (mm) | DWI | | | T1WI | | T2WI | | STIR | |
|  |  |  |  |  |  |  |  | Imaging  plane | ST  (mm) | B values (s/mm2) | Imaging  plane | ST  (mm) | Imaging  plane | ST  (mm) | Imaging  plane | ST  (mm) |
| Eschmann, 2007 | 1.5 | Siemens | Magnetom Avanto | Whole body | T2WSTIR-TSE/T1W-TSE/T1W FLASH 2D/T2W-TSE | 1 | NR | - | - | - | - | - | - | - | - | - |
| Lecouvet, 2007 | 1.5 | Philips | Achieva | Axial skeleton | T1WI/T2WI | 2 | 5 | - | - | - | S | 5-6/0.5-0.6 | S/C | 5-6/0.5-0.6 | - | - |
| Nemeth, 2007 | 1.5 | NR | NR | Whole body | DWI/T1WI/T2WI/FLAIR | NR | 5 | A | 5 | 0, 1000 | NR | 5 | NR | 5 | - | - |
| Venkitaraman, 2009 | 1.5 | NR | NR | Axial skeleton | T1WI/STIR | 1 | 8 | - | - | - | S | 8/1 | - | - | S | 8/1 |
| Venkitaraman, 2009# | 1.5 | Philips | Intera | Axial  skeleton | T1WI/STIR | 1 | 8 | - | - | - | C | 8 | - | - | C | 8 |
| Lecouvet, 2012 | 1.5 | Philips | Achieva | Whole body | DWI/T1WI/STIR | 2 | 5 | A | 5/0.5 | 0, 800 | C | 6/1 | - | - | C | 6/1 |
| Mosavi, 2012 | 1.5 | Philips | Intera | Whole body | DWI | 1 | 6 | A | 6/0 | 0, 1000 | - | - | - | - | - | - |
| Kitajima, 2014 | 1.5 or 3 | GE | Signa | Pelvis | DWI/T1WI/T2WI/ DCE | 1 | 2.5 | A | 5-7/0-1 | 0, 600, 1000 | A | 6/1 | A/C/S | 2.5-3/0-0.5 | - | - |
| Pasoglou, 2014 | 3 | NR | NR | Whole body | T1WI/DWI/PDFS | 3 | 1.2 | A | 5 | 0, 800 | C | 1.2 | - | - | - | - |
| Piccardo, 2014 | 1.5 | GE | Signa | Pelvis | STIR | 1 | 5 | - | - | - | - | - | - | - | A | 5/0.5 |
| Pasoglou, 2015 | 3 | Siemens | Verio | Whole body | DWI/T1WI | 2 | 1.2 | A | 5/0.5 | 0, 800 | C | 1.2/0 | - | - | - | - |
| Wieder, 2015 | 1.5 | Siemens | Magnetom Avanto | Whole body | T1-TSE/T2WI/DWI/STIR | 3 | 5 | A | 5 | 50, 500 | A | 5 | A/S/C | 5 | A | 5 |
| Barchetti, 2016 | 1.5 | Siemens | Magnetom Avanto | Whole body | T1WI/T2WI/STIR/DWI | 2 | 4 | A | 5 | 50, 500, 800/1000 | A/S | 5/4 | S | 5 | A/S | 5 |
| Conde-Moreno, 2016 | 1.5 | Siemens | Magnetom Avanto | Whole body | DWI/T1WI/STIR | 1 | 5 | A | 5 | 50, 900 | A | 5/NR | - | - | A/S | NR |
| Woo, 2016 | 3 | Philips | Intera | Pelvis | DWI/T1WI/T2WI/ DCE | 3 | 3 | A | 3.5/0 | 0, 1000 | A | 3-3.5/0 | A/C/S | 3-4/0-0.4 | - | - |
| Huysse, 2017 | NR | NR | NR | Axial skeleton | T1WI/T2WI/STIR | 2 | 3 | - | - | - | C | 5 | C | 5 | S | 3 |
| Vargas, 2017 | 1.5 or 3 | GE | NR | Pelvis | T1WI | 1 | 5 | - | - | - | A | 5/1 | - | - | - | - |
| Dyrberg, 2018 | 3 | Philips | Intera | Whole body | T1WI/STIR/DWI | 3 | NR | A | NR | NR | C/S | NR | - | - | C | NR |
| Larbi, 2018 | 1.5 | Philips | Achieva | Whole body | T1WI/STIR/DWI | 2 | 4 | A | 5 | 0, 800 | C | 4 | - | - | C | 4 |
| Zacho, 2018 | 3 | Philips | Intera | Whole body | T1WI/T2WI/DWI/STIR | 3 | 3 | A | 4 | 0, 600 | S/C | 4/3 | A | 4 | S/C | 4 |
| Johnston, 2019 | 3 | Philips | Intera | Whole body | modified Dixon/T2-TSE/DWI | 2 | 5 | A | 5 | 0, 100, 300, 1000 | - | - | A | 5 | - | - |
| MRI=Magnetic resonance imaging; T1WI=T1 weighted image; T2WI=T2 weighted image; STIR=Short tau inversion recovery; TSE=Turbo spin echo; FLASH=Fast low angle shot; DWI=Diffusion weighted imaging; FLAIR=Fluid attenuated inversion recovery; DCE=Dynamic Contrast Enhanced; PDFS=Proton density with fat signal suppression; ST=Slice thickness; A=Axial; C=Coronal; S=Sagittal; NR=Not reported; # shows different study | | | | | | | | | | | | | | | | |

| **eTable 7. Main characteristics of PET/CT in the included studies** | | | | | | | | |
| --- | --- | --- | --- | --- | --- | --- | --- | --- |
| Study, year | PET parameters | | | CT parameters | | | | Imaging  analysis |
|  | Contrast agent | Intravenous administration dose | Time between injection and image acquisition | Tube  Voltage  (kV) | Tube  current-time  product (mA) | Tube rotation  time (s) | ST  (mm) |  |
| Even, 2006 | 18F-NaF | 296-444 MBq | 60-90 min | 140 | 80 | 0.8 | NR | V + Semi |
| Beheshti, 2008 | 18F-Choline | 4.07 MBq/kg | 1 min | 140 | 80 | 0.5 | NR | V + Semi |
|  | 18F-NaF | 370-550 MBq | 60 min | 140 | 80 | 0.5 | NR | V + Semi |
| Beheshti, 2009 | 18F-Choline | 4.07 MBq/kg | 1 min | 140 | NR | 0.5 | 5 | V + Semi |
| Fuccio, 2010 | 11C-Choline | 370-555 MBq | 5 min | 120 | 60 | 0.8 | 5 | V + Semi |
| Iagaru, 2011 | 18F-NaF | 370 ± 37 MBq | 45 min | NR | NR | NR | NR | V |
|  | 18F-FDG | 555 ± 55.5 MBq | 60 min | NR | NR | NR | NR | V |
| Langsteger, 2011 | 18F-Choline | 4 MBq/kg | 10-20 min | NR | NR | NR | NR | V |
|  | 18F-NaF | 4 MBq/kg | 60-90 min | NR | NR | NR | NR | V |
| Bortot, 2012 | 18F-NaF | 370 MBq | 60 min | 130 | 50-80 | 0.8 | NR | NR |
| Jadvar, 2012 | 18F-FDG | 388.5 MBq | 60 min | 120 | 50 | NR | 5 | V + Semi |
|  | 18F-NaF | 525.4 MBq | 60 min | 120 | 50 | NR | 5 | V + Semi |
| Picchio, 2012 | 11C-Choline | 370 MBq | Immediately | NR | NR | NR | NR | V + Semi |
| Takesh, 2012 | 18F-Choline | 250 MBq | Immediately | 130 | 30 | NR | NR | V + Semi |
| Damle, 2013 | 18F-FDG | 370-555 MBq | 45 min | NR | NR | NR | NR | V |
|  | 18F-NaF | 370 MBq | 45-60 min | NR | NR | NR | NR | V |
| Kitajima, 2014 | 11C-Choline | 370-555 MBq | 5 min | 120 | 20-160 | 0.5 | 3.75 | V + Semi |
| Piccardo, 2014 | 18F-Choline | 3 MBq/kg | 10 min | 120 | 120-400 | 0.5 | 3.75/1.25 | V + Semi |
| Poulsen, 2014 | 18F-NaF | 3 MBq/kg | 60 min | 120 | 400 | NR | NR | V |
|  | 18F-Choline | 4 MBq/kg | 60 min | 120 | 400 | NR | NR | V |
| Evangelista, 2015 | 18F-Choline | 3 MBq/kg | 60 min | NR | NR | NR | NR | V + Semi |
| Sampath, 2015 | 18F-NaF/FDG | NR | NR | NR | NR | NR | NR | V + Semi |
| Wieder, 2015 | 11C-Choline | 600-900 MBq | 5 min | 120 | 240 | 0.5 | 5 | V + Semi |
| Conde-moreno, 2016 | 18F-Choline | 166.5-296 MBq | 45-60 min | NR | NR | NR | NR | V + Semi |
| Nanni, 2016 | 18F-FACBC | 370 MBq | NR | 120 | 80 | NR | NR | V + Semi |
|  | 11C-Choline | 3.4 MBq/kg | NR | 120 | 80 | NR | NR | V + Semi |
| Yi, 2016 | 13N-Ammonia | 555-740 MBq | 10 min | 140 | 180 | 0.5 | 5 | V + Semi |
|  | 18F-FDG | 5.18 MBq/kg | 45-60 min | 140 | 180 | 0.5 | 5 | V + Semi |
| Fonager, 2017 | 18F-NaF | 200 MBq | 30 min | 130 | 30 | NR | 3/5 | V |
| Huysse, 2017 | 18F-Choline | NR | NR | NR | NR | NR | NR | NR |
| Janssen, 2017 | 68Ga-PSMA | 120.3 MBq | 61.7 min | 120 | 30-40 | 0.4 | 5 | V + Semi |
| Kitajima, 2017 | 11C-Choline | 3.0 MBq/kg | 5 min | 120 | auto-100 | NR | 2 | V + Semi |
| Wondergem, 2017 | 18F-NaF | 189 MBq | 60 min | 110 | 25 | NR | 5 | V + Semi |
| Dyrberg, 2018 | 68Ga-PSMA | NR | NR | NR | NR | NR | NR | NR |
|  | 18F-NaF | NR | NR | NR | NR | NR | NR | NR |
| Kawanaka, 2018 | 11C-Choline | 3.0 MBq/kg | 6 min | 120 | 100 | NR | 2 | V + Semi |
| Lengana, 2018 | 68Ga-PSMA | Median 136.9 MBq (45.88-305.25) | 60 min | NR | NR | NR | NR | V + Semi |
| Zacho, 2018 | 68Ga-PSMA | 2 MBq/kg (100-200) | 60 min | 120 | 10-150 | NR | 0.625 | V |
|  | 18F-NaF | 200 MBq | 30 min | 120 | 10-150 | NR | 0.625 | V |
| Chen, 2019 | 18F-FACBC | 370 MBq | 5 min | 130 | 90 | 0.6 | NR | V + Semi |
| Johnston, 2019 | 18F-Choline | 327.4 MBq (198-410) | 60 min | 120 | 10 | 0.8 | NR | V |
| Uslu-Besli, 2019 | 68Ga-PSMA | 92.5-148 MBq | 45 min | 130 | 48-76 | 0.6 | 4 | V |
| PET/CT=Positron emission tomography/computed tomography; FDG=Fludeoxyglucose; NaF=Sodium fluoride; FACBC=Fluciclovine; PSMA=Prostate membrane antigen; V=Visual evaluation; Semi=Semi-quantitative evaluation; NR=Not reported | | | | | | | | |
|  |  |  |  |  |  |  |  |  |

| **eTable 8. Diagnostic data of the included studies on patient-based level** | | | | | | | |
| --- | --- | --- | --- | --- | --- | --- | --- |
| Study, year | Methods | Tracer or Tesla | Sensitive  (95% CI) | Specificity  (95% CI) | PLR  (95% CI) | NLR  (95% CI) | DOR  (95% CI) |
| Fuccio, 2010 | PET/CT | 11C-Choline | 0.86  (0.65, 0.97) | 1.00  (0.29, 1.00) | 6.78  (0.50, 91.19) | 0.17  (0.06, 0.49) | 39.00  (1.63, 932.30) |
| Picchio, 2012 | PET/CT | 11C-Choline | 0.89  (0.71, 0.98) | 0.98  (0.90, 1.00) | 45.33  (6.48, 317.12)2) | 0.11  (0.04, 0.33) | 400.00  (39.50, 4049.71)0) |
| Picchio, 2012* | PET/CT | 11C-Choline | 0.86  (0.42, 1.00) | 0.97  (0.85, 1.00) | 29.14  (4.13, 205.71)5) | 0.15  (0.02, 0.90) | 198.00  (10.80, 3616.91)0) |
| Kitajima, 2014 | PET/CT | 11C-Choline | 0.81  (0.54, 0.96) | 0.99  (0.93, 1.00) | 64.19  (9.03, 456.45)6) | 0.19  (0.07, 0.53) | 338.00  (32.62, 3502.20)0) |
| Nanni, 2016 | PET/CT | 11C-Choline | 0.83  (0.36, 1.00) | 1.00  (0.03, 1.00) | 3.14  (0.28, 35.75) | 0.29  (0.06, 1.46) | 11.00  (0.28, 433.80) |
| Kitajima, 2017 | PET/CT | 11C-Choline | 0.91  (0.59, 1.00) | 0.90  (0.56, 1.00) | 9.09  (1.40, 58.91) | 0.10  (0.02, 0.66) | 90.00  (4.88, 1659.50) |
| Kawanaka, 2018 | PET/CT | 11C-Choline | 0.94  (0.71, 1.00) | 0.92  (0.64, 1.00) | 12.24  (1.85, 80.73)) | 0.06  (0.01, 0.43) | 192.00  (10.87, 3390.10)0) |
| Yi, 2016 | PET/CT | 13N-Ammonia | 1.00  (0.74, 1.00) | 1.00  (0.77, 1.00) | 28.85  (1.89, 441.17)4) | 0.04  (0.01, 0.60) | 725.00  (13.38, 39278.11)70) |
| Langsteger, 2011 | PET/CT | 18F-Choline | 0.91  (0.71, 0.99) | 0.89  (0.65, 0.99) | 8.18  (2.20, 30.42) | 0.10  (0.03, 0.39) | 80.00  (10.12, 632.26) |
| Takesh, 2012 | PET/CT | 18F-Choline | 0.83  (0.59, 0.96) | 1.00  (0.82, 1.00) | 32.63  (2.10, 508.03)4) | 0.19  (0.07, 0.49) | 172.71  (8.28, 3601.01) |
| Evangelista, 2015 | PET/CT | 18F-Choline | 1.00  (0.72, 1.00) | 0.92  (0.78, 0.98) | 10.41  (3.81, 28.43)) | 0.05  (0.01, 0.69) | 226.71  (10.87, 4727.32)0) |
| Evangelista, 2015* | PET/CT | 18F-Choline | 1.00  (0.69, 1.00) | 0.86  (0.65, 0.97) | 6.27  (2.37, 16.60) | 0.05  (0.01, 0.81) | 117.00  (5.50, 2487.10) |
| Conde-Moreno, 2016 | PET/CT | 18F-Choline | 0.88  (0.64, 0.99) | 0.89  (0.65, 0.99) | 7.94  (2.13, 29.67) | 0.13  (0.04, 0.49) | 60.00  (7.48, 481.57) |
| Huysse, 2017 | PET/CT | 18F-Choline | 0.87  (0.60, 0.98) | 1.00  (0.93, 1.00) | 84.38  (5.31, 1341.17)10) | 0.16  (0.05, 0.49) | 534.60  (24.19, 11813.12)00) |
| Johnston, 2019 | PET/CT | 18F-Choline | 0.80  (0.28, 1.00) | 0.92  (0.64, 1.00) | 10.40  (1.50, 71.90)) | 0.22  (0.04, 1.26) | 48.00  (2.40, 958.25) |
| Nanni, 2016 | PET/CT | 18F-FACBC | 0.83  (0.36, 1.00) | 0.01  (0.01, 0.98) | 1.05  (0.43, 2.55) | 0.86  (0.05, 13.9) | 1.22  (0.03, 48.20) |
| Chen, 2019 | PET/CT | 18F-FACBC | 1.00  (0.77, 1.00) | 0.98  (0.92, 1.00) | 35.96  (10.55, 122.19)59) | 0.03  (0.01, 0.52) | 1049.80  (47.93, 2299.13)2.60) |
| Iagaru, 2011 | PET/CT | 18F-FDG | 0.56  (0.21, 0.86) | 1.00  (0.66, 1.00) | 11.00  (0.70, 173.67)6) | 0.47  (0.24, 0.95) | 23.22  (1.04, 517.94) |
| Jadvar, 2012 | PET/CT | 18F-FDG | 0.21  (0.05, 0.51) | 1.00  (0.85, 1.00) | 11.20  (0.62, 201.96)5) | 0.78  (0.59, 1.04) | 14.30  (0.68, 300.83) |
| Damle, 2013 | PET/CT | 18F-FDG | 0.72  (0.53, 0.86) | 1.00  (0.81, 1.00) | 25.64  (1.65, 397.62)8) | 0.30  (0.17, 0.51) | 86.58  (4.72, 1589.90) |
| Yi, 2016 | PET/CT | 18F-FDG | 1.00  (0.74, 1.00) | 0.86  (0.57, 0.98) | 5.77  (1.85, 17.98) | 0.05  (0.01, 0.71) | 125.00  (5.43, 2876.30) |
| Even, 2006 | PET/CT | 18F-NaF | 1.00  (0.85, 1.00) | 1.00  (0.84, 1.00) | 43.08  (2.78, 667.71)4) | 0.02  (0.01, 0.33) | 2021.00  (38.40, 1063.41)71.00) |
| Even, 2006* | PET/CT | 18F-NaF | 1.00  (0.75, 1.00) | 1.00  (0.72, 1.00) | 23.14  (1.53, 349.62)8) | 0.04  (0.01, 0.57) | 621.00  (11.40, 33839.11)90) |
| Iagaru, 2011 | PET/CT | 18F-NaF | 1.00  (0.66, 1.00) | 0.78  (0.40, 0.97) | 3.80  (1.29, 11.22) | 0.07  (0.01, 1.02) | 57.00  (2.36, 1375.80) |
| Langsteger, 2011 | PET/CT | 18F-NaF | 0.91  (0.71, 0.99) | 0.83  (0.59, 0.96) | 5.46  (1.93, 15.45) | 0.11  (0.03, 0.42) | 50.00  (7.40, 337.77) |
| Bortot, 2012 | PET/CT | 18F-NaF | 0.50  (0.01, 0.99) | 1.00  (0.59, 1.00) | 8.00  (0.44, 147.24)) | 0.53  (0.17, 1.68) | 15.00  (0.39, 576.70) |
| Jadvar, 2012 | PET/CT | 18F-NaF | 0.43  (0.18, 0.71) | 0.83  (0.61, 0.95) | 2.46  (0.84, 7.23) | 0.69  (0.42, 1.13) | 3.56  (0.79, 16.14) |
| Mosavi, 2012 | PET/CT | 18F-NaF | 1.00  (0.66, 1.00) | 0.81  (0.64, 0.92) | 4.69  (2.44, 9.02) | 0.06  (0.01, 0.94) | 74.73  (3.89, 1434.50) |
| Damle, 2013 | PET/CT | 18F-NaF | 1.00  (0.89, 1.00) | 0.71  (0.44, 0.90) | 3.22  (1.60, 6.48) | 0.02  (0.01, 0.35) | 147.73  (7.60, 2872.70) |
| Fonager, 2017 | PET/CT | 18F-NaF | 0.89  (0.71, 0.98) | 0.90  (0.56, 1.00) | 8.89  (1.38, 57.34) | 0.12  (0.04, 0.37) | 72.00  (6.60, 785.24) |
| Wondergem, 2017 | PET/CT | 18F-NaF | 1.00  (0.94, 1.00) | 0.98  (0.88, 1.00) | 29.10  (6.04, 140.26)7) | 0.01  (0.01, 0.13) | 3485.00  (138.63, 876.15)08.30) |
| Dyrberg, 2018 | PET/CT | 18F-NaF | 0.95  (0.75, 1.00) | 0.97  (0.85, 1.00) | 33.25  (4.81, 230.01)8) | 0.05  (0.01, 0.35) | 646.00  (38.20, 10926.16)00) |
| Zacho, 2018 | PET/CT | 18F-NaF | 0.90  (0.56, 1.00) | 0.98  (0.91, 1.00) | 51.30  (7.27, 361.83)9) | 0.1  (0.020, 0.65) | 504.00  (28.87, 8798.82)0) |
| Sampath, 2015 | PET/CT | 18F-NaF/FDG | 1.00  (0.85, 1.00) | 0.75  (0.48, 0.93) | 3.70  (1.67, 8.18) | 0.03  (0.01, 0.47) | 125.00  (6.21, 2516.80) |
| Janssen, 2017 | PET/CT | 68Ga-PSMA | 1.00  (0.88, 1.00) | 1.00  (0.86, 1.00) | 51.13  (3.28, 796.21)1) | 0.02  (0.01, 0.27) | 3009.00  (57.61, 1571.18)51.60) |
| Dyrberg, 2018 | PET/CT | 68Ga-PSMA | 1.00  (0.83, 1.00) | 1.00  (0.90, 1.00) | 70.29  (4.48, 1103.09)10) | 0.02  (0.01, 0.37) | 2911.00  (55.63, 1523.34)16.70) |
| Lengana, 2018 | PET/CT | 68Ga-PSMA | 0.96  (0.80, 1.00) | 1.00  (0.96, 1.00) | 166.22  (10.46, 2602.23)40.60) | 0.06  (0.01, 0.27) | 2975.00  (117.58, 752.27)72.20) |
| Zacho, 2018 | PET/CT | 68Ga-PSMA | 0.80  (0.44, 0.98) | 1.00  (0.94, 1.00) | 91.18  (5.67, 1467.23)70) | 0.23  (0.08, 0.68) | 397.80  (17.56, 9012.73)0) |
| Uslu-Besli, 2019 | PET/CT | 68Ga-PSMA | 0.91  (0.59, 1.00) | 1.00  (0.81, 1.00) | 31.50  (2.03, 488.53)2) | 0.13  (0.03, 0.58) | 245.00  (9.12, 6581.70) |
| Lecouvet, 2007 | MRI | 1.5 | 1.00  (0.91, 1.00) | 0.88  (0.69, 0.98) | 7.34  (2.77, 19.46) | 0.01  (0.01, 0.22) | 533.57  (26.37, 10795.12)50) |
| Nemeth, 2007 | MRI | 1.5 | 0.14  (0.01, 0.58) | 1.00  (0.03, 1.00) | 0.75  (0.05, 12.34) | 1.08  (0.46, 2.58) | 0.69  (0.02, 26.91) |
| Venkitaraman, 2009 | MRI | 1.5 | 0.86  (0.57, 0.98) | 0.98  (0.92, 1.00) | 36.43  (9.11, 145.62)9) | 0.15  (0.04, 0.53) | 249.00  (32.01, 1936.83)0) |
| Venkitaraman, 2009# | MRI | 1.5 | 0.70  (0.35, 0.93) | 1.00  (0.88, 1.00) | 40.91  (2.54, 658.11)7) | 0.32  (0.14, 0.77) | 126.43  (5.87, 2722.60) |
| Lecouvet, 2012 | MRI | 1.5 | 0.99  (0.91, 1.00) | 0.99  (0.91, 1.00) | 97.04  (6.16, 1529.12)80) | 0.01  (0.01, 0.16) | 9797.00  (190.59, 503.15)594.90) |
| Mosavi, 2012 | MRI | 1.5 | 0.56  (0.21, 0.86) | 0.94  (0.81, 0.99) | 10.00  (2.30, 43.41)) | 0.47  (0.23, 0.98) | 21.25  (3.06, 147.83) |
| Kitajima, 2014 | MRI | 1.5/3 | 0.88  (0.62, 0.98) | 0.96  (0.89, 0.99) | 23.04  (7.48, 70.99)) | 0.13  (0.04, 0.48) | 177.33  (27.12, 1159.68)0) |
| Pasoglou, 2014 | MRI | 3 | 1.00  (0.66, 1.00) | 1.00  (0.84, 1.00) | 41.80  (2.69, 649.80)4) | 0.05  (0.01, 0.76) | 817.00  (15.06, 44322.81)80) |
| Pasoglou, 2015 | MRI | 3 | 1.00  (0.69, 1.00) | 1.00  (0.83, 1.00) | 40.09  (2.58, 621.91)2) | 0.05  (0.01, 0.70) | 861.00  (15.93, 46535.16)90) |
| Barchetti, 2016 | MRI | 1.5 | 0.99  (0.92, 1.00) | 0.96  (0.90, 0.99) | 26.94  (8.87, 81.84)) | 0.02  (0.01, 0.10) | 1817.00  (184.72, 178.12)73.00) |
| Conde-Moreno, 2016 | MRI | 1.5 | 0.71  (0.44, 0.90) | 0.72  (0.47, 0.90) | 2.54  (1.14, 5.69) | 0.41  (0.19, 0.90) | 6.24  (1.44, 27.10) |
| Woo, 2016 | MRI | 3 | 0.95  (0.76, 1.00) | 0.99  (0.97, 1.00) | 91.11  (29.44, 281.12)97) | 0.05  (0.01, 0.33) | 1893.30  (188.28, 190.17)39.10) |
| Huysse, 2017 | MRI | NR | 1.00  (0.78, 1.00) | 0.96  (0.86, 1.00) | 19.38  (5.77, 65.07)) | 0.03  (0.01, 0.50) | 589.00  (26.80, 12944.22)90) |
| Vargas, 2017 | MRI | 1.5/3 | 0.78  (0.65, 0.88) | 0.98  (0.95, 1.00) | 44.50  (14.38, 137.02)76) | 0.22  (0.14, 0.36) | 199.36  (54.19, 733.48) |
| Dyrberg, 2018 | MRI | 3 | 0.80  (0.56, 0.94) | 0.83  (0.66, 0.93) | 4.67  (2.18, 9.99) | 0.24  (0.10, 0.59) | 19.33  (4.75, 78.77) |
| Larbi, 2018 | MRI | 1.5 | 1.00  (0.91, 1.00) | 1.00  (0.75, 1.00) | 27.63  (1.82, 420.31)5) | 0.01  (0.01, 0.22) | 2025.00  (38.26, 1071.10)85.90) |
| Zacho, 2018 | MRI | 3 | 0.25  (0.03, 0.65) | 0.92  (0.82, 0.98) | 3.25  (0.71, 14.94) | 0.81  (0.54, 1.22) | 4.00  (0.60, 26.68) |
| Johnston, 2019 | MRI | 3 | 0.90  (0.37, 1.00) | 0.89  (0.59, 0.99) | 7.80  (1.68, 36.14) | 0.11  (0.01, 1.58) | 69.00  (2.35, 2028.80) |
| CI=Confidence interval; PLR=Positive likelihood ratio; NLR=Negative likelihood ratio; DOR=Diagnostic odds ratio; PET/CT=Positron emission tomography/computed tomography; MRI=Magnetic resonance imaging; FACBC=Fluciclovine; FDG=Fludeoxyglucose; NaF=Sodium fluoride; PSMA=Prostate membrane antigen; NR=Not reported; * shows different diagnostic data from one study; # shows different study | | | | | | | |
|  |  |  |  |  |  |  |  |
|  |  |  |  |  |  |  |  |

| **eTable 9. Diagnostic data of the included studies on lesion-based level** | | | | | | | |
| --- | --- | --- | --- | --- | --- | --- | --- |
| Study, year | Methods | Tracer or Tesla | Sensitive  (95% CI) | Specificity  (95% CI) | PLR  (95% CI) | NLR  (95% CI) | DOR  (95% CI) |
| Eschmann, 2007 | PET/CT | 11C-Choline | 0.92  (0.80, 0.98) | 0.50  (0.01, 1.00) | 1.84  (0.26, 13.12) | 0.16  (0.02, 1.41) | 11.86  (0.20, 693.56)) |
| Wieder, 2015 | PET/CT | 11C-Choline | 0.93  (0.84, 0.98) | 0.98  (0.97, 0.99) | 59.74  (26.93, 132.42)9) | 0.07  (0.03, 0.17) | 823.33  (244.14, 2745.56)76.60) |
| Beheshti, 2008 | PET/CT | 18F-Choline | 0.74  (0.67, 0.80) | 1.00  (0.97, 1.00) | 202.55  (12.72, 322.23)6.20) | 0.26  (0.21, 0.33) | 773.97  (47.25, 1262.34)78.30) |
| Beheshti, 2008* | PET/CT | 18F-Choline | 0.66  (0.58, 0.74) | 0.96  (0.92, 0.99) | 17.11  (7.74, 37.80) | 0.35  (0.28, 0.44) | 48.69  (20.14, 117.72)5) |
| Beheshti, 2010 | PET/CT | 18F-Choline | 0.79  (0.73, 0.84) | 0.97  (0.91, 0.99) | 25.97  (8.51, 79.29) | 0.22  (0.17, 0.28) | 118.29  (36.11, 387.23)44) |
| Piccardo, 2014 | PET/CT | 18F-Choline | 0.93  (0.66, 1.00) | 0.92  (0.62, 1.00) | 11.14  (1.70, 73.18) | 0.08  (0.01, 0.52) | 143.00  (7.98, 2562.56)20) |
| Poulsen, 2014 | PET/CT | 18F-Choline | 0.85  (0.80, 0.89) | 0.91  (0.86, 0.95) | 9.50  (5.75, 15.69) | 0.17  (0.13, 0.22) | 56.39  (30.06, 105.75)9) |
| Even, 2006 | PET/CT | 18F-NaF | 1.00  (0.94, 1.00) | 1.00  (0.96, 1.00) | 198.28  (12.49, 314.23)8.40) | 0.01  (0.01, 0.14) | 22885.00  (448.04, 53489.03)1168930.00) |
| Even, 2006* | PET/CT | 18F-NaF | 1.00  (0.91, 1.00) | 1.00  (0.95, 1.00) | 142.29  (8.98, 2253.10)40) | 0.01  (0.01, 0.19) | 11869.00  (231.16, 31984.20)609406.70) |
| Beheshti, 2008 | PET/CT | 18F-NaF | 0.81  (0.75, 0.87) | 0.93  (0.87, 0.96) | 11.03  (6.05, 20.11) | 0.20  (0.15, 0.28) | 54.00  (25.72, 113.33)6) |
| Beheshti, 2008* | PET/CT | 18F-NaF | 0.77  (0.69, 0.83) | 0.46  (0.38, 0.54) | 1.42  (1.20, 1.68) | 0.51  (0.36, 0.71) | 2.80  (1.71, 4.59) |
| Poulsen, 2014 | PET/CT | 18F-NaF | 0.93  (0.90, 0.96) | 0.54  (0.46, 0.62) | 2.02  (1.71, 2.40) | 0.13  (0.09, 0.19) | 15.86  (9.53, 26.41) |
| Janssen, 2017 | PET/CT | 68Ga-PSMA | 0.97  (0.94, 0.99) | 1.00  (0.75, 1.00) | 27.20  (1.79, 413.69)) | 0.03  (0.01, 0.07) | 915.00  (46.74, 1790.45)11.70) |
| Eschmann, 2007 | MRI | 1.5 | 0.88  (0.75, 0.96) | 0.50  (0.01, 1.00) | 1.76  (0.25, 12.50) | 0.24  (0.03, 2.02) | 7.18  (0.13, 400.10) |
| Piccardo, 2014 | MRI | 1.5 | 1.00  (0.77, 1.00) | 0.92  (0.62, 1.00) | 8.38  (1.85, 37.85) | 0.04  (0.01, 0.58) | 222.33  (8.26, 5985.34)20) |
| Pasoglou, 2015 | MRI | 3 | 0.66  (0.49, 0.80) | 1.00  (0.84, 1.00) | 28.81  (1.84, 450.17)) | 0.35  (0.23, 0.54) | 81.55  (4.60, 1445.70)0) |
| Wieder, 2015 | MRI | 1.5 | 0.79  (0.67, 0.88) | 0.88  (0.84, 0.91) | 6.27  (4.72, 8.33) | 0.25  (0.16, 0.38) | 25.60  (13.47, 48.63)) |
| Huysse, 2017 | MRI | NR | 1.00  (0.86, 1.00) | 0.93  (0.82, 0.98) | 12.20  (5.02, 29.63) | 0.02  (0.01, 0.34) | 560.78  (29.03, 1080.56)33.60) |
| CI=Confidence interval; PLR=Positive likelihood ratio; NLR=Negative likelihood ratio; DOR=Diagnostic odds ratio; PET/CT=Positron emission tomography/computed tomography; MRI=Magnetic resonance imaging; NaF=Sodium fluoride; PSMA=Prostate membrane antigen; NR=Not reported; * shows different diagnostic data | | | | | | | |
|  |  |  |  |  |  |  |  |

| **eTable 10. The results of quality assessment of the included studies** | | | | | | | | | | | | |
| --- | --- | --- | --- | --- | --- | --- | --- | --- | --- | --- | --- | --- |
| Study, year | 1 | 2 | 3 | 4 | 5 | 6 | 7 | 8 | 9 | 10 | 11 | QUADAS-2 |
| Even, 2006 | U | Y | Y | Y | U | Y | U | Y | Y | Y | Y | 8 |
| Eschmann, 2007 | Y | Y | Y | U | U | Y | Y | U | Y | Y | Y | 8 |
| Lecouvet, 2007 | U | Y | Y | Y | U | Y | Y | Y | Y | Y | Y | 9 |
| Nemeth, 2007 | Y | Y | Y | Y | U | Y | Y | Y | Y | Y | Y | 10 |
| Beheshti, 2008 | U | Y | Y | Y | Y | Y | U | Y | Y | Y | Y | 9 |
| Beheshti, 2009 | U | Y | Y | Y | Y | Y | U | Y | Y | Y | Y | 9 |
| Venkitaraman, 2009 | Y | Y | Y | Y | U | Y | U | Y | Y | Y | Y | 9 |
| Venkitaraman, 2009# | Y | Y | Y | Y | U | Y | U | Y | Y | Y | Y | 9 |
| Fuccio, 2010 | U | Y | U | U | Y | Y | Y | U | Y | Y | Y | 7 |
| Iagaru, 2011 | Y | Y | Y | Y | Y | Y | Y | Y | N | N | N | 8 |
| Langsteger, 2011 | Y | Y | Y | Y | N | Y | Y | Y | Y | N | Y | 9 |
| Bortot, 2012 | Y | Y | Y | Y | U | Y | Y | Y | Y | Y | Y | 10 |
| Jadvar, 2012 | Y | Y | Y | Y | Y | Y | Y | Y | Y | Y | Y | 11 |
| Lecouvet, 2012 | Y | Y | U | Y | U | Y | Y | Y | Y | Y | Y | 9 |
| Mosavi, 2012 | Y | Y | U | Y | U | Y | Y | Y | Y | Y | Y | 9 |
| Picchio, 2012 | Y | Y | U | Y | U | Y | Y | Y | Y | Y | Y | 9 |
| Takesh, 2012 | U | Y | U | Y | U | Y | Y | Y | Y | Y | Y | 8 |
| Damle, 2013 | U | Y | Y | Y | U | Y | Y | Y | Y | Y | Y | 9 |
| Kitajima, 2014 | U | Y | Y | Y | Y | Y | Y | Y | Y | Y | Y | 10 |
| Pasoglou, 2014 | U | Y | U | Y | U | Y | Y | Y | Y | Y | Y | 8 |
| Piccardo, 2014 | U | Y | U | Y | Y | Y | U | Y | Y | Y | Y | 8 |
| Poulsen, 2014 | Y | Y | Y | Y | U | Y | Y | U | Y | Y | Y | 9 |
| Evangelista, 2015 | Y | Y | Y | Y | Y | Y | Y | Y | Y | Y | Y | 11 |
| Pasoglou, 2015 | Y | Y | U | Y | U | Y | Y | Y | Y | Y | Y | 9 |
| Sampath, 2015 | Y | Y | U | Y | U | Y | Y | Y | Y | Y | Y | 9 |
| Wieder, 2015 | Y | Y | Y | Y | Y | Y | Y | Y | Y | N | Y | 10 |
| Barchetti, 2016 | Y | Y | Y | Y | U | Y | Y | U | Y | Y | Y | 9 |
| Conde-moreno, 2016 | Y | Y | Y | Y | Y | Y | Y | Y | Y | Y | Y | 11 |
| Nanni, 2016 | U | Y | U | U | Y | Y | Y | Y | Y | Y | Y | 8 |
| Woo, 2016 | Y | Y | U | Y | U | Y | U | Y | Y | Y | Y | 8 |
| Yi, 2016 | Y | Y | Y | Y | Y | Y | Y | Y | Y | Y | Y | 11 |
| Fonager, 2017 | Y | Y | U | Y | U | Y | Y | Y | Y | Y | Y | 9 |
| Huysse, 2017 | U | Y | Y | Y | Y | Y | Y | Y | Y | Y | Y | 10 |
| Janssen, 2017 | U | Y | Y | Y | U | Y | U | Y | Y | Y | Y | 8 |
| Kitajima, 2017 | Y | Y | U | Y | Y | Y | U | Y | Y | Y | Y | 9 |
| Vargas, 2017 | Y | Y | Y | Y | U | Y | Y | Y | Y | Y | Y | 10 |
| Wondergem, 2017 | Y | Y | Y | Y | U | Y | Y | Y | Y | Y | Y | 10 |
| Dyrberg, 2018 | Y | Y | Y | Y | Y | Y | Y | Y | Y | Y | N | 10 |
| Kawanaka, 2018 | Y | Y | Y | Y | Y | Y | Y | Y | Y | N | Y | 10 |
| Larbi, 2018 | Y | Y | Y | Y | Y | Y | Y | Y | Y | N | Y | 10 |
| Lengana, 2018 | Y | Y | Y | Y | N | Y | Y | Y | Y | N | Y | 9 |
| Zacho, 2018 | Y | Y | Y | Y | Y | Y | Y | Y | N | N | N | 8 |
| Chen, 2019 | Y | Y | Y | Y | Y | Y | Y | Y | Y | N | Y | 10 |
| Johnston, 2019 | Y | Y | N | Y | N | Y | Y | Y | Y | N | Y | 8 |
| Uslu-besll, 2019 | Y | Y | Y | Y | N | Y | Y | Y | Y | N | Y | 9 |
| 1. Was a consecutive or random sample of patients enrolled? | | | | | | | | | | | | |
| 2. Was a case-control design avoided? | | | | | | | | | | | | |
| 3. Did the study avoid inappropriate exclusions? | | | | | | | | | | | | |
| 4. Were the index test results interpreted without knowledge of the results of the reference standard? | | | | | | | | | | | | |
| 5. If a threshold was used, was it pre-specified? | | | | | | | | | | | | |
| 6. Is the reference standard likely to correctly classify the target condition? | | | | | | | | | | | | |
| 7. Were the reference standard results interpreted without knowledge of the results of the index test? | | | | | | | | | | | | |
| 8. Was there an appropriate interval between index test (s) and reference standard? | | | | | | | | | | | | |
| 9. Did all patients receive a reference standard? | | | | | | | | | | | | |
| 10. Did all patients receive the same reference standard? | | | | | | | | | | | | |
| 11. Were all patients included in the analysis? | | | | | | | | | | | | |
| # shows different study | | | | | | | | | | | | |

| **eTable 11. PET/CT with different tracers for detecting bone metastasis in patients with PCa by deleting studies with only one diagnostic test** | | | | | | | | |
| --- | --- | --- | --- | --- | --- | --- | --- | --- |
| Test | Absolute Sensitivity | Absolute Specificity | Diagnostic OR  [Rank] | Superiority Index [Rank] | Relative Sensitivity | Relative Specificity | Datasets, n | Studies, n |
| 18F-NaF | 0.90  (0.65-0.99) | 0.79  (0.48-0.94) | 99.26  (4.67-459.42)  [4] | 4.22  (0.33-9.00)  [2] | 1.00  (1.00-1.00) | 1.00  (1.00-1.00) | 5 | 5 |
| 18F-Choline | 0.72  (0.23-0.99) | 0.71  (0.25-0.98) | 97.94  (0.35-609.59)  [5] | 1.87  (0.11-9.00)  [4] | 0.81  (0.26-1.20) | 0.91  (0.32-1.45) | 2 | 2 |
| 11C-Choline | 0.66  (0.18-0.98) | 0.72  (0.19-1.00) | 30862.69  (0.20-16257.20)  [3] | 1.89  (0.11-9.00)  [3] | 0.74  (0.20-1.19) | 0.93  (0.24-1.65) | 1 | 1 |
| 68Ga-PSMA | 0.78  (0.37-0.99) | 0.87  (0.42-1.00) | 1273929.50  (1.58-409239)  [1] | 4.73  (0.20-11.00)  [1] | 0.87  (0.41-1.25) | 1.14  (0.53-1.83) | 1 | 1 |
| 18F-FDG | 0.55  (0.19-0.85) | 0.88  (0.46-1.00) | 50792.23  (0.60-59328.18)  [2] | 1.87  (0.11-7.00)  [4] | 0.61  (0.22-0.99) | 1.15  (0.57-1.81) | 2 | 2 |
| 18F-FACBC | 0.66  (0.17-0.98) | 0.32  (0.00-0.79) | 7.49  (0.00-43.78)  [6] | 0.40  (0.09-3.00)  [6] | 0.74  (0.19-1.20) | 0.43  (0.00-1.14) | 1 | 1 |
| Data are reported as mean (range) unless otherwise indicated.  PET/CT=Positron emission tomography/computed tomography; PCa=Prostate cancer; NaF=Sodium fluoride; PSMA=Prostate membrane antigen; FDG=Fludeoxyglucose; FACBC=Fluciclovine; OR=Odds ratio | | | | | | | | |

| **eTable 12. PET/CT with different tracers for detecting bone metastasis in patients with PCa by deleting studies with a QUADAS-2 score of 7** | | | | | | | | |
| --- | --- | --- | --- | --- | --- | --- | --- | --- |
| Test | Absolute Sensitivity | Absolute Specificity | Diagnostic OR  [Rank] | Superiority Index [Rank] | Relative Sensitivity | Relative Specificity | Datasets, n | Studies, n |
| 18F-NaF | 0.95  (0.83-0.99) | 0.88  (0.75-0.95) | 244.30  (32.19-823.73)  [2] | 3.25  (0.20-9.00)  [2] | 1.00  (1.00-1.00) | 1.00  (1.00-1.00) | 10 | 10 |
| 18F-Choline | 0.89  (0.75-0.95) | 0.91  (0.78-0.97) | 120.16  (20.86-353.50)  [4] | 1.71  (0.14-7.00)  [4] | 0.94  (0.79-1.08) | 1.04  (0.87-1.23) | 7 | 7 |
| 11C-Choline | 0.85  (0.67-0.95) | 0.93  (0.75-0.99) | 193.16  (15.04-756.68)  [3] | 1.98  (0.14-7.00)  [3] | 0.90  (0.71-1.06) | 1.06  (0.85-1.27) | 6 | 6 |
| 68Ga-PSMA | 0.91  (0.66-0.99) | 0.98  (0.76-1.00) | 12474212.25  (37.66-4819013)  [1] | 7.18  (0.33-11.00)  [1] | 0.96  (0.69-1.13) | 1.11  (0.87-1.33) | 5 | 5 |
| 18F-FDG | 0.68  (0.34-0.92) | 0.86  (0.46-0.99) | 79.37  (1.26-487.34)  [6] | 0.56  (0.09-3.00)  [5] | 0.72  (0.36-0.99) | 0.98  (0.52-1.24) | 3 | 3 |
| 18F-FACBC | 0.79  (0.37-0.99) | 0.57  (0.16-0.95) | 79.64  (0.36-475.75)  [5] | 0.50  (0.09-3.00)  [6] | 0.84  (0.39-1.11) | 0.65  (0.18-1.11) | 2 | 2 |
| Data are reported as mean (range) unless otherwise indicated.  PET/CT=Positron emission tomography/computed tomography; PCa=Prostate cancer; NaF=Sodium fluoride; PSMA=Prostate membrane antigen; FDG=Fludeoxyglucose; FACBC=Fluciclovine; OR=Odds ratio | | | | | | | | |

| **eTable 13. PET/CT with different tracers for detecting bone metastasis in patients with PCa by deleting study with the maximum sample size** | | | | | | | | |
| --- | --- | --- | --- | --- | --- | --- | --- | --- |
| Test | Absolute Sensitivity | Absolute Specificity | Diagnostic OR  [Rank] | Superiority Index [Rank] | Relative Sensitivity | Relative Specificity | Datasets, n | Studies, n |
| 18F-NaF | 0.95  (0.84-0.99) | 0.88  (0.77-0.95) | 248.15  (34.14-799.75)  [2] | 3.33  (0.20-9.00)  [2] | 1.00  (1.00-1.00) | 1.00  (1.00-1.00) | 9 | 9 |
| 18F-Choline | 0.89  (0.78-0.95) | 0.91  (0.78-0.97) | 123.18  (21.09-361.86)  [4] | 1.71  (0.14-7.00)  [4] | 0.94  (0.82-1.07) | 1.03  (0.86-1.20) | 6 | 6 |
| 11C-Choline | 0.86  (0.73-0.94) | 0.94  (0.79-0.99) | 208.88  (21.37-780.88)  [3] | 1.92  (0.14-7.00)  [3] | 0.91  (0.77-1.05) | 1.07  (0.88-1.24) | 6 | 6 |
| 68Ga-PSMA | 0.91  (0.69-0.98) | 0.99  (0.85-1.00) | 3379817.37  (49.99-5941029)  [1] | 7.30  (0.60-11.00)  [1] | 0.96  (0.72-1.11) | 1.12  (0.96-1.30) | 4 | 4 |
| 18F-FDG | 0.69  (0.34-0.92) | 0.85  (0.46-0.99) | 81.17  (1.35-497.30)  [6] | 0.49  (0.09-3.00)  [6] | 0.73  (0.36-0.99) | 0.96  (0.51-1.22) | 2 | 2 |
| 18F-FACBC | 0.80  (0.37-0.99) | 0.59  (0.18-0.96) | 92.40  (0.40-598.84)  [5] | 0.55  (0.09-3.00)  [5] | 0.84  (0.39-1.09) | 0.67  (0.20-1.10) | 2 | 2 |
| Data are reported as mean (range) unless otherwise indicated.  PET/CT=Positron emission tomography/computed tomography; PCa=Prostate cancer; NaF=Sodium fluoride; PSMA=Prostate membrane antigen; FDG=Fludeoxyglucose; FACBC=Fluciclovine; OR=Odds ratio | | | | | | | | |

| **eTable 14. PET/CT with different tracers for detecting bone metastasis in patients with PCa by deleting study with the minimum sample size** | | | | | | | | |
| --- | --- | --- | --- | --- | --- | --- | --- | --- |
| Test | Absolute Sensitivity | Absolute Specificity | Diagnostic OR  [Rank] | Superiority Index [Rank] | Relative Sensitivity | Relative Specificity | Datasets, n | Studies, n |
| 18F-NaF | 0.94  (0.80-0.99) | 0.89  (0.75-0.96) | 236.10  (28.87-804.55)  [2] | 3.57  (0.20-9.00)  [2] | 1.00  (1.00-1.00) | 1.00  (1.00-1.00) | 9 | 9 |
| 18F-Choline | 0.89  (0.74-0.96) | 0.90  (0.71-0.97) | 128.13  (15.70-420.30)  [4] | 2.00  (0.14-7.00)  [4] | 0.95  (0.78-1.12) | 1.02  (0.80-1.21) | 6 | 6 |
| 11C-Choline | 0.85  (0.65-0.94) | 0.93  (0.73-0.99) | 195.36  (14.43-711.27)  [3] | 2.30  (0.14-7.00)  [3] | 0.91  (0.70-1.09) | 1.06  (0.82-1.25) | 6 | 6 |
| 68Ga-PSMA | 0.89  (0.57-0.99) | 0.95  (0.56-1.00) | 3339723.23  (9.27-2503536)  [1] | 6.18  (0.20-11.00)  [1] | 0.95  (0.60-1.16) | 1.07  (0.60-1.30) | 4 | 4 |
| 18F-FDG | 0.72  (0.33-0.97) | 0.77  (0.38-0.99) | 88.05  (0.77-578.72)  [6] | 0.69  (0.09-5.00)  [5] | 0.77  (0.35-1.06) | 0.88  (0.42-1.19) | 2 | 2 |
| 18F-FACBC | 0.80  (0.37-0.99) | 0.57  (0.17-0.96) | 115.77  (0.35-593.32)  [5] | 0.64  (0.09-5.00)  [6] | 0.86  (0.38-1.14) | 0.64  (0.19-1.09) | 2 | 2 |
| Data are reported as mean (range) unless otherwise indicated.  PET/CT=Positron emission tomography/computed tomography; PCa=Prostate cancer; NaF=Sodium fluoride; PSMA=Prostate membrane antigen; FDG=Fludeoxyglucose; FACBC=Fluciclovine; OR=Odds ratio | | | | | | | | |

| **eTable 15. PET/CT with different tracers for detecting bone metastasis in patients with PCa by detecting studies published before 2010** | | | | | | | | |
| --- | --- | --- | --- | --- | --- | --- | --- | --- |
| Test | Absolute Sensitivity | Absolute Specificity | Diagnostic OR  [Rank] | Superiority Index [Rank] | Relative Sensitivity | Relative Specificity | Datasets, n | Studies, n |
| 18F-NaF | 0.94  (0.81-0.98) | 0.85  (0.71-0.94) | 156.79  (22.39-513.99)  [3] | 2.70  (0.20-7.00)  [2] | 1.00  (1.00-1.00) | 1.00  (1.00-1.00) | 8 | 8 |
| 18F-Choline | 0.88  (0.76-0.95) | 0.91  (0.77-0.97) | 116.81  (20.53-358.71)  [4] | 1.91  (0.14-7.00)  [4] | 0.94  (0.80-1.11) | 1.07  (0.88-1.29) | 7 | 7 |
| 11C-Choline | 0.86  (0.73-0.94) | 0.94  (0.79-0.99) | 203.90  (20.39-738.00)  [2] | 2.26  (0.20-7.00)  [3] | 0.92  (0.77-1.09) | 1.11  (0.90-1.34) | 7 | 7 |
| 68Ga-PSMA | 0.89  (0.63-0.98) | 0.98  (0.78-1.00) | 2805379.69  (36.15-4640164)  [1] | 6.88  (0.60-11.00)  [1] | 0.96  (0.67-1.16) | 1.15  (0.91-1.38) | 5 | 5 |
| 18F-FDG | 0.68  (0.35-0.92) | 0.85  (0.48-0.99) | 80.23  (1.26-497.24)  [6] | 0.53  (0.09-3.00)  [6] | 0.73  (0.37-1.00) | 1.00  (0.56-1.31) | 3 | 3 |
| 18F-FACBC | 0.79  (0.36-0.99) | 0.57  (0.18-0.96) | 84.68  (0.37-498.68)  [5] | 0.58  (0.09-3.00)  [5] | 0.85  (0.38-1.12) | 0.67  (0.21-1.14) | 2 | 2 |
| Data are reported as mean (range) unless otherwise indicated.  PET/CT=Positron emission tomography/computed tomography; PCa=Prostate cancer; NaF=Sodium fluoride; PSMA=Prostate membrane antigen; FDG=Fludeoxyglucose; FACBC=Fluciclovine; OR=Odds ratio | | | | | | | | |

| **eTable 16. PET/CT with different tracers for detecting bone metastasis in patients with PCa based on clinical settings of cancers** | | | | | | | | | |
| --- | --- | --- | --- | --- | --- | --- | --- | --- | --- |
|  | Test | Absolute Sensitivity | Absolute Specificity | Diagnostic OR  [Rank] | Superiority Index [Rank] | Relative Sensitivity | Relative Specificity | Datasets, n | Studies, n |
| Newly | 18F-NaF | 0.77  (0.33-0.99) | 0.73  (0.30-0.97) | 210.98  (0.68-1149.75)  [3] | 1.24  (0.20-5.00)  [3] | 1.00  (1.00-1.00) | 1.00  (1.00-1.00) | 2 | 2 |
|  | 18F-Choline | 0.82  (0.44-0.99) | 0.82  (0.49-0.96) | 259.70  (2.08-1634.16)  [2] | 1.95  (0.20-5.00)  [1] | 1.16  (0.54-2.49) | 1.23  (0.64-2.68) | 3 | 3 |
|  | 68Ga-PSMA | 0.77  (0.30-1.00) | 0.74  (0.30-1.00) | 33385279.77  (0.50-998155)  [1] | 1.82  (0.20-5.00)  [2] | 1.08  (0.37-2.33) | 1.13  (0.38-2.74) | 1 | 1 |
|  | | | | | | | | | |
| Mixed | 18F-NaF | 0.92  (0.69-0.99) | 0.84  (0.62-0.95) | 197.64  (9.19-978.86)  [3] | 3.89  (0.20-9.00)  [2] | 1.00  (1.00-1.00) | 1.00  (1.00-1.00) | 7 | 7 |
|  | 18F-Choline | 0.74  (0.26-0.99) | 0.71  (0.23-0.98) | 130.70  (0.40-797.97)  [4] | 1.32  (0.09-7.00)  [4] | 0.81  (0.28-1.19) | 0.86  (0.28-1.30) | 1 | 1 |
|  | 11C-Choline | 0.77  (0.36-0.98) | 0.77  (0.27-0.99) | 216.95  (0.93-1177.60)  [2] | 1.67  (0.11-9.00)  [3] | 0.85  (0.38-1.23) | 0.93  (0.33-1.35) | 2 | 2 |
|  | 68Ga-PSMA | 0.87  (0.50-0.99) | 0.93  (0.52-1.00) | 1798143.21  (5.00-2123755)  [1] | 6.39  (0.20-11.00)  [1] | 0.96  (0.55-1.31) | 1.12  (0.61-1.53) | 3 | 3 |
|  | 18F-FDG | 0.67  (0.34-0.91) | 0.85  (0.47-0.99) | 79.64  (1.32-492.47)  [5] | 1.23  (0.11-7.00)  [5] | 0.73  (0.37-1.09) | 1.03  (0.56-1.43) | 3 | 3 |
|  | 18F-FACBC | 0.78  (0.37-0.99) | 0.56  (0.17-0.94) | 61.83  (0.33-350.89)  [6] | 0.77  (0.09-5.00)  [6] | 0.86  (0.41-1.22) | 0.67  (0.20-1.19) | 1 | 1 |
|  | | | | | | | | | |
| Treated | 18F-NaF | 0.73  (0.22-0.99) | 0.77  (0.25-1.00) | 873.49  (0.42-3946.46)  [2] | 1.62  (0.14-7.00)  [3] | 1.00  (1.00-1.00) | 1.00  (1.00-1.00) | 1 | 1 |
|  | 18F-Choline | 0.81  (0.53-0.94) | 0.84  (0.46-0.99) | 190.26  (2.60-1143.45)  [4] | 2.07  (0.14-7.00)  [2] | 1.32  (0.64-3.67) | 1.28  (0.56-3.31) | 3 | 3 |
|  | 11C-Choline | 0.85  (0.66-0.94) | 0.90  (0.58-0.99) | 209.14  (7.05-1041.87)  [3] | 3.04  (0.20-7.00)  [1] | 1.39  (0.77-3.87) | 1.38  (0.75-3.50) | 5 | 5 |
|  | 68Ga-PSMA | 0.65  (0.19-0.97) | 0.76  (0.30-1.00) | 113806.95  (0.34-41194.94)  [1] | 1.24  (0.14-7.00)  [4] | 1.06  (0.25-3.03) | 1.16  (0.37-3.14) | 1 | 1 |
| Data are reported as mean (range) unless otherwise indicated.  PET/CT=Positron emission tomography/computed tomography; PCa=Prostate cancer; NaF=Sodium fluoride; PSMA=Prostate membrane antigen; FDG=Fludeoxyglucose; FACBC=Fluciclovine; OR=Odds ratio | | | | | | | | | |

| **eTable 17. PET/CT with different trances for detecting bone metastasis in patients with PCa based on numbers of patients** | | | | | | | | | |
| --- | --- | --- | --- | --- | --- | --- | --- | --- | --- |
|  | Test | Absolute Sensitivity | Absolute Specificity | Diagnostic OR  [Rank] | Superiority Index [Rank] | Relative Sensitivity | Relative Specificity | Datasets, n | Studies, n |
| No. of patients  < 50 | 18F-NaF | 0.92  (0.69-0.99) | 0.83  (0.65-0.93) | 150.83  (9.88-639.34)  [2] | 2.48  (0.14-7.00)  [2] | 1.00  (1.00-1.00) | 1.00  (1.00-1.00) | 7 | 7 |
|  | 18F-Choline | 0.88  (0.67-0.97) | 0.88  (0.69-0.96) | 109.87  (11.67-407.30)  [4] | 2.69  (0.20-7.00)  [1] | 0.96  (0.72-1.31) | 1.08  (0.83-1.38) | 5 | 5 |
|  | 11C-Choline | 0.83  (0.50-0.97) | 0.85  (0.54-0.99) | 144.01  (3.39-770.87)  [3] | 2.09  (0.14-7.00)  [4] | 0.91  (0.53-1.26) | 1.04  (0.63-1.40) | 3 | 3 |
|  | 68Ga-PSMA | 0.73  (0.25-0.99) | 0.83  (0.36-1.00) | 169045.35  (0.54-333962)  [1] | 2.36  (0.11-9.00)  [3] | 0.79  (0.26-1.21) | 1.02  (0.43-1.44) | 1 | 1 |
|  | 18F-FDG | 0.68  (0.34-0.92) | 0.86  (0.49-0.99) | 76.80  (1.45-451.12)  [5] | 1.03  (0.11-5.00)  [5] | 0.74  (0.37-1.09) | 1.05  (0.58-1.42) | 3 | 3 |
|  | | | | | | | | | |
| No. of patients  ≥ 50 | 18F-NaF | 0.88  (0.52-0.99) | 0.91  (0.59-0.99) | 1010.88  (6.49-6596.84)  [2] | 2.99  (0.14-7.00)  [2] | 1.00  (1.00-1.00) | 1.00  (1.00-1.00) | 3 | 3 |
|  | 18F-Choline | 0.73  (0.31-0.96) | 0.83  (0.39-1.00) | 321.14  (0.93-2269.25)  [3] | 1.04  (0.11-7.00)  [4] | 0.86  (0.36-1.50) | 0.93  (0.42-1.44) | 2 | 2 |
|  | 11C-Choline | 0.78  (0.46-0.94) | 0.92  (0.58-1.00) | 238.93  (3.92-1502.46)  [4] | 1.44  (0.14-7.00)  [3] | 0.91  (0.51-1.56) | 1.02  (0.63-1.56) | 4 | 4 |
|  | 68Ga-PSMA | 0.88  (0.55-0.99) | 0.98  (0.77-1.00) | 4221304.82  (20.52-4198172)  [1] | 5.60  (0.33-9.00)  [1] | 1.03  (0.62-1.68) | 1.09  (0.84-1.64) | 4 | 4 |
|  | 18F-FDG | 0.79  (0.35-0.99) | 0.56  (0.16-0.95) | 97.13  (0.32-477.81)  [5] | 0.56  (0.11-3.00)  [5] | 0.92  (0.40-1.59) | 0.63  (0.18-1.12) | 2 | 2 |
| Data are reported as mean (range) unless otherwise indicated.  PET/CT=Positron emission tomography/computed tomography; PCa=Prostate cancer; NaF=Sodium fluoride; PSMA=Prostate membrane antigen; FDG=Fludeoxyglucose; OR=Odds ratio | | | | | | | | | |

| **eTable 18. PET/CT with different tracers for detecting bone metastasis in patients with PCa based on ages of patients** | | | | | | | | | | | | | | | | | | |  |
| --- | --- | --- | --- | --- | --- | --- | --- | --- | --- | --- | --- | --- | --- | --- | --- | --- | --- | --- | --- |
|  | Test | | Absolute Sensitivity | | Absolute Specificity | | Diagnostic OR  [Rank] | | Superiority Index [Rank] | | | Relative Sensitivity | Relative Specificity | | Datasets, n | | Studies, n | |  |
| Age 60-70 | 18F-NaF | | 0.89  (0.57-0.99) | | 0.78  (0.50-0.93) | | 102.28  (4.09-506.17)  [4] | | 2.77  (0.14-7.00)  [2] | | | 1.00  (1.00-1.00) | 1.00  (1.00-1.00) | | 4 | | 4 | |  |
|  | 18F-Choline | | 0.81  (0.52-0.95) | | 0.85  (0.51-0.98) | | 90.38  (3.41-461.03)  [5] | | 1.90  (0.14-7.00)  [4] | | | 0.93  (0.58-1.47) | 1.11  (0.66-1.74) | | 3 | | 3 | |  |
|  | 11C-Choline | | 0.79  (0.49-0.94) | | 0.91  (0.58-1.00) | | 236.94  (3.88-1407.62)  [3] | | 2.41  (0.14-9.00)  [3] | | | 0.91  (0.54-1.43) | 1.20  (0.73-1.83) | | 4 | | 4 | |  |
|  | 68Ga-PSMA | | 0.88  (0.62-0.98) | | 0.96  (0.65-1.00) | | 8361852.00  (11.37-2265086)  [1] | | 6.97  (0.33-11.00)  [1] | | | 1.01  (0.68-1.57) | 1.26  (0.82-1.92) | | 4 | | 4 | |  |
|  | 18F-FDG | | 0.58  (0.15-0.92) | | 0.80  (0.33-1.00) | | 66248.31  (0.30-47775.65)  [2] | | 1.02  (0.09-7.00)  [5] | | | 0.67  (0.17-1.21) | 1.06  (0.41-1.79) | | 1 | | 1 | |  |
|  | 18F-FACBC | | 0.68  (0.20-0.99) | | 0.31  (0.00-0.83) | | 10.71  (0.00-58.54)  [6] | | 0.32  (0.09-1.00)  [6] | | | 0.79  (0.22-1.39) | 0.41  (0.00-1.14) | | 1 | | 1 | |  |
|  | | | | | | | | | | | | | | | | | | |  |
| Age  > 70 | 18F-NaF | | 0.90  (0.63-0.99) | | 0.94  (0.74-0.99) | | 995.24  (18.00-5819.48)  [4] | | 5.24  (0.20-11.00)  [1] | | | 1.00  (1.00-1.00) | 1.00  (1.00-1.00) | | 5 | | 5 | |  |
|  | 18F-Choline | | 0.85  (0.48-0.99) | | 0.84  (0.57-0.96) | | 199.99  (3.96-1104.62)  [5] | | 1.93  (0.11-7.00)  [4] | | | 0.96  (0.52-1.41) | 0.90  (0.61-1.17) | | 3 | | 3 | |  |
|  | 11C-Choline | | 0.81  (0.47-0.97) | | 0.86  (0.53-0.99) | | 148.70  (2.91-829.41)  [6] | | 1.74  (0.11-9.00)  [5] | | | 0.92  (0.51-1.35) | 0.91  (0.56-1.21) | | 3 | | 3 | |  |
|  | 68Ga-PSMA | | 0.76  (0.33-1.00) | | 0.78  (0.32-1.00) | | 43889776.11  (0.64-1912703.9)  [1] | | 2.61  (0.09-11.00)  [2] | | | 0.86  (0.36-1.34) | 0.83  (0.34-1.19) | | 1 | | 1 | |  |
|  | 18F-FDG | | 0.76  (0.29-1.00) | | 0.68  (0.20-0.98) | | 503744.81  (0.35-41276.09)  [3] | | 1.23  (0.09-7.00)  [6] | | | 0.86  (0.32-1.37) | 0.73  (0.21-1.10) | | 1 | | 1 | |  |
|  | 18F-FACBC | | 0.74  (0.28-1.00) | | 0.82  (0.29-1.00) | | 13663567.15  (0.59-132467.37)  [2] | | 2.45  (0.09-11.00)  [3] | | | 0.83  (0.31-1.36) | 0.87  (0.31-1.23) | | 1 | | 1 | |  |
| Data are reported as mean (range) unless otherwise indicated.  PET/CT=Positron emission tomography/computed tomography; PCa=Prostate cancer; NaF=Sodium fluoride; PSMA=Prostate membrane antigen; FDG=Fludeoxyglucose; FACBC=Fluciclovine; OR=Odds ratio | | | | | | | | | | | | | | | | | | |  |
| **eTable 19. PET/CT with different tracers for detecting bone metastasis in patients with PCa based on continents of studies.** | | | | | | | | | | | | | | | | | | | |
|  | | Test | | Absolute Sensitivity | | Absolute Specificity | | Diagnostic OR  [Rank] | | Superiority Index [Rank] | Relative Sensitivity | | | Relative Specificity | | Datasets, n | | Studies, n | |
| Europe | | 18F-NaF | | 0.93  (0.79-0.98) | | 0.91  (0.78-0.97) | | 298.44  (32.22-1039.70)  [3] | | 2.30  (0.20-5.00)  [2] | 1.00  (1.00-1.00) | | | 1.00  (1.00-1.00) | | 8 | | 8 | |
|  |  | 18F-Choline | | 0.88  (0.73-0.95) | | 0.91  (0.77-0.97) | | 118.71  (19.95-371.09)  [4] | | 1.00  (0.14-5.00)  [3] | 0.95  (0.79-1.13) | | | 1.00  (0.84-1.17) | | 7 | | 7 | |
|  |  | 11C-Choline | | 0.81  (0.51-0.95) | | 0.89  (0.53-1.00) | | 326.10  (3.81-2051.49)  [2] | | 0.89  (0.14-5.00)  [4] | 0.87  (0.54-1.09) | | | 0.98  (0.58-1.20) | | 4 | | 4 | |
|  |  | 68Ga-PSMA | | 0.87  (0.52-0.99) | | 0.96  (0.68-1.00) | | 1227836.31  (10.62-3423275)  [1] | | 3.59  (0.20-7.00)  [1] | 0.93  (0.58-1.14) | | | 1.06  (0.74-1.26) | | 4 | | 4 | |
|  | |  | |  | |  | |  | |  |  | | |  | |  | |  | |
| Outside Europe | | 18F-NaF | | 0.85  (0.42-1.00) | | 0.61  (0.21-0.90) | | 84975.22  (0.60-62282.15)  [2] | | 1.26  (0.14-5.00)  [4] | 1.00  (1.00-1.00) | | | 1.00  (1.00-1.00) | | 2 | | 2 | |
|  |  | 11C-Choline PET/CT | | 0.79  (0.45-0.96) | | 0.87  (0.55-0.99) | | 130.90  (3.09-697.09)  [3] | | 2.44  (0.20-7.00)  [1] | 0.99  (0.50-1.93) | | | 1.67  (0.82-4.30) | | 3 | | 3 | |
|  |  | 18F-FDG PET/CT | | 0.67  (0.33-0.92) | | 0.84  (0.48-0.99) | | 68.94  (1.33-431.08)  [4] | | 1.38  (0.14-5.00)  [3] | 0.84  (0.37-1.75) | | | 1.61  (0.68-4.05) | | 3 | | 3 | |
|  |  | 18F-FACBC PET/CT | | 0.78  (0.32-1.00) | | 0.76  (0.27-0.99) | | 214951.31  (0.54-125349.87)  [1] | | 2.12  (0.14-7.00)  [2] | 0.97  (0.35-1.91) | | | 1.43  (0.42-3.62) | | 1 | | 1 | |
| Data are reported as mean (range) unless otherwise indicated.  PET/CT=Positron emission tomography/computed tomography; PCa=Prostate cancer; NaF=Sodium fluoride; PSMA=Prostate membrane antigen; FDG=Fludeoxyglucose; FACBC=Fluciclovine; OR=Odds ratio | | | | | | | | | | | | | | | | | | | |

| **eTable 20. PET/CT with different tracers for detecting bone metastasis in patients with PCa based on study design** | | | | | | | | | |
| --- | --- | --- | --- | --- | --- | --- | --- | --- | --- |
|  | Test | Absolute Sensitivity | Absolute Specificity | Diagnostic OR  [Rank] | Superiority Index [Rank] | Relative Sensitivity | Relative Specificity | Datasets, n | Studies, n |
| Prospective | 18F-NaF | 0.95  (0.81-0.99) | 0.85  (0.67-0.94) | 235.95  (19.60-912.73)  [2] | 4.21  (0.43-9.00)  [2] | 1.00  (1.00-1.00) | 1.00  (1.00-1.00) | 8 | 8 |
|  | 18F-Choline | 0.84  (0.59-0.95) | 0.87  (0.59-0.98) | 94.84  (6.44-395.67)  [4] | 2.08  (0.14-7.00)  [3] | 0.89  (0.62-1.08) | 1.04  (0.71-1.33) | 4 | 4 |
|  | 11C-Choline | 0.79  (0.37-0.98) | 0.78  (0.34-0.99) | 231.14  (1.17-1357.61)  [3] | 1.53  (0.11-7.00)  [4] | 0.83  (0.39-1.08) | 0.93  (0.40-1.33) | 2 | 2 |
|  | 68Ga-PSMA | 0.88  (0.56-0.99) | 0.96  (0.64-1.00) | 940803.15  (11.47-2437131)  [1] | 6.71  (0.33-11.00)  [1] | 0.93  (0.58-1.13) | 1.14  (0.74-1.47) | 4 | 4 |
|  | 18F-FDG | 0.68  (0.33-0.92) | 0.86  (0.48-0.99) | 79.68  (1.39-494.82)  [5] | 1.02  (0.11-5.00)  [5] | 0.72  (0.34-1.00) | 1.02  (0.55-1.37) | 3 | 3 |
|  | 18F-FACBC | 0.68  (0.20-0.99) | 0.30  (0.00-0.84) | 12.27  (0.00-80.58)  [6] | 0.27  (0.09-1.00)  [6] | 0.72  (0.21-1.08) | 0.35  (0.00-0.98) | 1 | 1 |
|  | | | | | | | | | |
| Retrospective | 18F-NaF | 0.78  (0.38-0.99) | 0.83  (0.40-0.99) | 318.73  (1.18-2630.31)  [3] | 1.97  (0.11-7.00)  [5] | 1.00  (1.00-1.00) | 1.00  (1.00-1.00) | 2 | 2 |
|  | 18F-Choline | 0.83  (0.46-0.99) | 0.83  (0.47-0.98) | 150.42  (1.98-1025.02)  [5] | 1.98  (0.11-7.00)  [4] | 1.15  (0.53-2.27) | 1.06  (0.52-2.16) | 3 | 3 |
|  | 11C-Choline | 0.82  (0.52-0.94) | 0.93  (0.69-0.99) | 219.13  (8.26-1120.10)  [4] | 3.08  (0.20-9.00)  [1] | 1.14  (0.64-2.20) | 1.19  (0.79-2.31) | 5 | 5 |
|  | 68Ga-PSMA | 0.72  (0.21-0.99) | 0.79  (0.31-1.00) | 112854.15  (0.44-140311.19)  [2] | 1.99  (0.11-9.00)  [3] | 1.00  (0.28-2.20) | 1.01  (0.38-2.12) | 1 | 1 |
|  | 18F-FACBC | 0.77  (0.31-1.00) | 0.76  (0.28-0.99) | 284962.09  (0.59-203378.16)  [1] | 2.18  (0.11-9.00)  [2] | 1.08  (0.36-2.26) | 0.98  (0.32-2.05) | 1 | 1 |
| Data are reported as mean (range) unless otherwise indicated.  PET/CT=Positron emission tomography/computed tomography; PCa=Prostate cancer; NaF=Sodium fluoride; PSMA=Prostate membrane antigen; FDG=Fludeoxyglucose; FACBC=Fluciclovine; OR=Odds ratio | | | | | | | | | |

| **eTable 21. PET/CT with different tracers for detecting bone metastasis in patients with PCa based on methods of imaging analyses** | | | | | | | | | |
| --- | --- | --- | --- | --- | --- | --- | --- | --- | --- |
|  | Test | Absolute Sensitivity | Absolute Specificity | Diagnostic OR  [Rank] | Superiority Index [Rank] | Relative Sensitivity | Relative Specificity | Datasets, n | Studies, n |
| V | 18F-NaF | 0.89  (0.66-0.98) | 0.79  (0.53-0.93) | 68.21  (5.68-274.06)  [4] | 2.18  (0.20-5.00)  [2] | 1.00  (1.00-1.00) | 1.00  (1.00-1.00) | 5 | 5 |
|  | 18F-Choline | 0.78  (0.38-0.97) | 0.80  (0.41-0.98) | 72.51  (1.24-435.00)  [3] | 1.55  (0.14-5.00)  [3] | 0.88  (0.43-1.22) | 1.03  (0.51-1.50) | 2 | 2 |
|  | 68Ga-PSMA | 0.77  (0.35-0.97) | 0.86  (0.43-1.00) | 48592.01  (1.63-158272)  [2] | 2.43  (0.14-7.00)  [1] | 0.87  (0.41-1.25) | 1.12  (0.53-1.67) | 2 | 2 |
|  | 18F-FDG | 0.54  (0.15-0.85) | 0.87  (0.42-1.00) | 67594.40  (0.54-45719.72)  [1] | 0.99  (0.14-5.00)  [4] | 0.61  (0.17-1.01) | 1.12  (0.52-1.66) | 2 | 2 |
|  | | | | | | | | | |
| V + Semi | 18F-NaF | 0.92  (0.48-1.00) | 0.92  (0.56-1.00) | 11697378.12  (6.85-7807231)  [2] | 5.75  (0.14-11.00)  [1] | 1.00  (1.00-1.00) | 1.00  (1.00-1.00) | 3 | 3 |
|  | 18F-Choline | 0.86  (0.55-0.98) | 0.87  (0.59-0.97) | 114.76  (5.95-544.40)  [5] | 1.38  (0.11-7.00)  [4] | 0.98  (0.57-1.80) | 0.97  (0.64-1.59) | 4 | 4 |
|  | 11C-Choline | 0.86  (0.71-0.94) | 0.94  (0.78-0.99) | 197.76  (18.93-751.83)  [4] | 1.97  (0.14-7.00)  [3] | 0.98  (0.74-1.79) | 1.05  (0.82-1.70) | 7 | 7 |
|  | 68Ga-PSMA | 0.88  (0.46-1.00) | 0.91  (0.46-1.00) | 23450171.83  (3.99-4579568)  [1] | 4.75  (0.14-11.00)  [2] | 1.00  (0.48-1.85) | 1.01  (0.48-1.73) | 2 | 2 |
|  | 18F-FDG | 0.79  (0.32-1.00) | 0.69  (0.20-0.98) | 103072.88  (0.38-95102.20)  [3] | 1.19  (0.09-7.00)  [5] | 0.90  (0.33-1.81) | 0.77  (0.22-1.38) | 1 | 1 |
|  | 18F-FACBC | 0.80  (0.37-0.99) | 0.58  (0.17-0.96) | 93.95  (0.37-705.56)  [6] | 0.52  (0.09-3.00)  [6] | 0.91  (0.39-1.76) | 0.64  (0.18-1.26) | 2 | 2 |
| Data are reported as mean (range) unless otherwise indicated.  PET/CT=Positron emission tomography/computed tomography; PCa=Prostate cancer; V=Visual evaluation; Semi=semi-quantitative evaluation; NaF=Sodium fluoride; PSMA=Prostate membrane antigen; FDG=Fludeoxyglucose; FACBC=Fluciclovine; OR=Odds ratio | | | | | | | | | |

| **eTable 22. PET/CT with different tracers and MRI with different field strength for detecting bone metastasis in patients with PCa** | | | | | | | | |
| --- | --- | --- | --- | --- | --- | --- | --- | --- |
| Test | Absolute Sensitivity | Absolute Specificity | Diagnostic OR  [Rank] | Superiority Index [Rank] | Relative Sensitivity | Relative Specificity | Datasets, n | Studies, n |
| 18F-NaF PET/CT | 0.95  (0.91-0.99) | 0.89  (0.84-0.94) | 267.82  (36.99-847.43)  [2] | 2.47  (0.14-7.00)  [2] | 1.00  (1.00-1.00) | 1.00  (1.00-1.00) | 10 | 9 |
| 18F-Choline PET/CT | 0.89  (0.84-0.94) | 0.91  (0.86-0.96) | 130.71  (21.62-390.64)  [5] | 1.29  (0.11-7.00)  [5] | 0.93  (0.87-0.99) | 1.03  (0.95-1.11) | 7 | 7 |
| 11C-Choline PET/CT | 0.86  (0.81-0.91) | 0.94  (0.89-0.99) | 193.74  (18.78-734.78)  [4] | 1.45  (0.11-7.00)  [4] | 0.91  (0.84-0.98) | 1.06  (0.97-1.15) | 7 | 7 |
| 68Ga-PSMA PET/CT | 0.91  (0.83-0.99) | 0.97  (0.90-1.04) | 8528534.31  (22.28-5335418)  [1] | 6.65  (0.14-11.00)  [1] | 0.96  (0.86-1.06) | 1.10  (1.00-1.20) | 5 | 5 |
| 1.5-T MRI | 0.82  (0.73-0.91) | 0.92  (0.87-0.97) | 95.19  (11.30-334.70)  [6] | 0.77  (0.09-5.00)  [6] | 0.86  (0.76-0.96) | 1.04  (0.96-1.12) | 8 | 8 |
| 3.0-T MRI | 0.89  (0.80-0.98) | 0.88  (0.79-0.97) | 232.53  (8.93-1231.80)  [3] | 1.76  (0.09-9.00)  [3] | 0.94  (0.84-1.04) | 1.00  (0.88-1.12) | 5 | 5 |
| Data are reported as mean (range) unless otherwise indicated.  PET/CT=Positron emission tomography/computed tomography; MRI=Magnetic resonance imaging; PCa=Prostate cancer; NaF=Sodium fluoride; PSMA=Prostate membrane antigen; T=Tesla; OR=Odds ratio | | | | | | | | |

| **eTable 23. PET/CT with different tracers and MRI with different numbers of sequences for detecting bone metastasis in patients with PCa** | | | | | | | | |
| --- | --- | --- | --- | --- | --- | --- | --- | --- |
| Test | Absolute Sensitivity | Absolute Specificity | Diagnostic OR  [Rank] | Superiority Index [Rank] | Relative Sensitivity | Relative Specificity | Datasets, n | Studies, n |
| 18F-NaF PET/CT | 0.95  (0.91-0.99) | 0.89  (0.84-0.94) | 274.82  (37.05-925.50)  [2] | 2.28  (0.14-7.00)  [2] | 1.00  (1.00-1.00) | 1.00  (1.00-1.00) | 10 | 9 |
| 18F-Choline PET/CT | 0.89  (0.84-0.94) | 0.92  (0.87-0.97) | 133.63  (23.52-395.25)  [5] | 1.25  (0.11-7.00)  [5] | 0.94  (0.87-1.01) | 1.03  (0.95-1.11) | 7 | 7 |
| 11C-Choline PET/CT | 0.86  (0.81-0.91) | 0.94  (0.89-0.99) | 217.10  (21.84-806.23)  [3] | 1.68  (0.11-7.00)  [4] | 0.91  (0.84-0.98) | 1.06  (0.98-1.14) | 7 | 7 |
| 68Ga-PSMA PET/CT | 0.91  (0.83-0.99) | 0.97  (0.90-1.04) | 1743915.56  (29.90-5009362)  [1] | 6.55  (0.20-11.00)  [1] | 0.95  (0.85-1.05) | 1.09  (0.99-1.19) | 5 | 5 |
| Multi-sequence  MRI | 0.91  (0.87-0.95) | 0.93  (0.90-0.96) | 188.33  (39.52-509.39)  [4] | 2.04  (0.14-7.00)  [3] | 0.95  (0.89-1.01) | 1.05  (0.98-1.12) | 14 | 14 |
| Single-sequence MRI | 0.64  (0.48-0.80) | 0.89  (0.76-1.02) | 69.40  (1.21-363.69)  [6] | 0.36  (0.09-1.02)  [6] | 0.67  (0.50-0.84) | 1.00  (0.84-1.16) | 2 | 2 |
| Data are reported as mean (range) unless otherwise indicated.  PET/CT=Positron emission tomography/computed tomography; MRI=Magnetic resonance imaging; PCa=Prostate cancer; NaF=Sodium fluoride; PSMA=Prostate membrane antigen; OR=Odds ratio | | | | | | | | |

| **eTable 24. PET/CT with different tracers and MRI with/without DWI for detecting bone metastasis in patients with PCa** | | | | | | | | |
| --- | --- | --- | --- | --- | --- | --- | --- | --- |
| Test | Absolute Sensitivity | Absolute Specificity | Diagnostic OR  [Rank] | Superiority Index [Rank] | Relative Sensitivity | Relative Specificity | Datasets, n | Studies, n |
| 18F-NaF PET/CT | 0.95  (0.91-0.99) | 0.89  (0.84-0.94) | 272.03  (37.71-889.59)  [4] | 1.81  (0.14-7.00)  [3] | 1.00  (1.00-1.00) | 1.00  (1.00-1.00) | 10 | 9 |
| 18F-Choline PET/CT | 0.89  (0.84-0.94) | 0.91  (0.86-0.96) | 119.04  (20.89-344.78)  [6] | 0.72  (0.09-5.00)  [6] | 0.93  (0.86-1.00) | 1.02  (0.94-1.10) | 7 | 7 |
| 11C-Choline PET/CT | 0.86  (0.81-0.91) | 0.94  (0.89-0.99) | 209.43  (21.02-759.07)  [5] | 1.05  (0.11-5.00)  [5] | 0.91  (0.84-0.98) | 1.06  (0.97-1.15) | 7 | 7 |
| 68Ga-PSMA PET/CT | 0.90  (0.81-0.99) | 0.98  (0.92-1.04) | 2887469.96  (40.59-5359604)  [1] | 5.84  (0.20-11.00)  [1] | 0.95  (0.85-1.05) | 1.11  (1.02-1.20) | 5 | 5 |
| DWI used  MRI | 0.94  (0.89-0.99) | 0.93  (0.88-0.98) | 575.84  (42.76-2275.10)  [2] | 3.63  (0.14-9.00)  [2] | 0.99  (0.92-1.06) | 1.05  (0.97-1.13) | 8 | 8 |
| No-DWI MRI | 0.86  (0.71-1.01) | 0.86  (0.74-0.98) | 373.18  (4.05-2274.81)  [3] | 1.44  (0.09-9.00)  [4] | 0.91  (0.75-1.07) | 0.97  (0.82-1.12) | 3 | 3 |
| Data are reported as mean (range) unless otherwise indicated.  PET/CT=Positron emission tomography/computed tomography; MRI=Magnetic resonance imaging; PCa=Prostate cancer; DWI=Diffusion weighted imaging; NaF=Sodium fluoride; PSMA=Prostate membrane antigen; OR=Odds ratio | | | | | | | | |

| **eTable 25. PET/CT with different tracers and MRI with different numbers of imaging planes for detecting bone metastasis in patients with PCa** | | | | | | | | |
| --- | --- | --- | --- | --- | --- | --- | --- | --- |
| Test | Absolute Sensitivity | Absolute Specificity | Diagnostic OR  [Rank] | Superiority Index [Rank] | Relative Sensitivity | Relative Specificity | Datasets, n | Studies, n |
| 18F-NaF PET/CT | 0.95  (0.91-0.99) | 0.89  (0.84-0.94) | 260.50  (34.82-873.54)  [3] | 1.62  (0.14-7.00)  [3] | 1.00  (1.00-1.00) | 1.00  (1.00-1.00) | 10 | 9 |
| 18F-Choline PET/CT | 0.89  (0.84-0.94) | 0.91  (0.86-0.96) | 128.22  (22.69-379.67)  [5] | 0.97  (0.11-5.00)  [5] | 0.94  (0.87-1.01) | 1.03  (0.94-1.12) | 7 | 7 |
| 11C-Choline PET/CT | 0.86  (0.81-0.91) | 0.94  (0.89-0.99) | 202.30  (20.58-757.59)  [4] | 1.36  (0.11-5.00)  [4] | 0.91  (0.84-0.98) | 1.06  (0.97-1.15) | 7 | 7 |
| 68Ga-PSMA PET/CT | 0.91  (0.82-1.00) | 0.98  (0.92-1.04) | 2325426.38  (35.49-5480643)  [1] | 6.40  (0.20-11.00)  [1] | 0.96  (0.86-1.06) | 1.11  (1.01-1.21) | 5 | 5 |
| ≥2 imaging planes MRI | 0.95  (0.90-1.00) | 0.93  (0.89-0.97) | 703.91  (52.55-2724.70)  [2] | 4.27  (0.20-9.00)  [2] | 1.01  (0.94-1.08) | 1.06  (0.98-1.14) | 10 | 10 |
| 1 imaging plane MRI | 0.75  (0.69-0.81) | 0.92  (0.86-0.98) | 53.01  (8.08-153.77)  [6] | 0.25  (0.09-1.00)  [6] | 0.80  (0.72-0.88) | 1.04  (0.95-1.13) | 6 | 6 |
| Data are reported as mean (range) unless otherwise indicated.  PET/CT=Positron emission tomography/computed tomography; MRI=Magnetic resonance imaging; PCa=Prostate cancer; NaF=Sodium fluoride; PSMA=Prostate membrane antigen; OR=Odds ratio | | | | | | | | |

| **eTable 26. PET/CT with different tracers and MRI with different coverage for detecting bone metastasis in patients with PCa** | | | | | | | | |
| --- | --- | --- | --- | --- | --- | --- | --- | --- |
| Test | Absolute Sensitivity | Absolute Specificity | Diagnostic OR  [Rank] | Superiority Index [Rank] | Relative Sensitivity | Relative Specificity | Datasets, n | Studies, n |
| 18F-NaF PET/CT | 0.95  (0.85-0.99) | 0.89  (0.76-0.95) | 269.31  (37.85-898.86)  [2] | 1.88  (0.14-7.00)  [2] | 1.00  (1.00-1.00) | 1.00  (1.00-1.00) | 10 | 9 |
| 18F-Choline PET/CT | 0.89  (0.78-0.95) | 0.92  (0.80-0.97) | 131.84  (27.37-372.34)  [5] | 1.09  (0.11-5.00)  [5] | 0.94  (0.82-1.07) | 1.04  (0.90-1.23) | 7 | 7 |
| 11C-Choline PET/CT | 0.87  (0.76-0.94) | 0.95  (0.83-0.99) | 241.06  (27.21-826.89)  [4] | 1.65  (0.11-7.00)  [3] | 0.91  (0.79-1.05) | 1.08  (0.92-1.26) | 7 | 7 |
| 68Ga-PSMA PET/CT | 0.92  (0.73-0.99) | 0.98  (0.79-1.00) | 3844738.92  (45.21-5571377)  [1] | 7.27  (0.33-11.00)  [1] | 0.97  (0.76-1.11) | 1.11  (0.88-1.31) | 5 | 5 |
| Axial skeleton or WB MRI | 0.84  (0.70-0.93) | 0.93  (0.86-0.96) | 89.86  (22.64-228.31)  [6] | 0.58  (0.09-3.00)  [6] | 0.88  (0.73-1.02) | 1.05  (0.94-1.22) | 14 | 14 |
| Pelvis MRI | 0.82  (0.53-0.95) | 0.94  (0.69-0.99) | 252.60  (7.04-1050.20)  [3] | 1.41  (0.09-7.00)  [4] | 0.86  (0.56-1.04) | 1.07  (0.79-1.26) | 3 | 3 |
| Data are reported as mean (range) unless otherwise indicated.  PET/CT=Positron emission tomography/computed tomography; MRI=Magnetic resonance imaging; PCa=Prostate cancer; NaF=Sodium fluoride; PSMA=Prostate membrane antigen; WB=Whole body; OR=Odds ratio | | | | | | | | |

| **eTable 27. PET/CT with different tracers and MRI with different field strength for detecting bone metastasis in patients with PCa by deleting studies with only one diagnostic test** | | | | | | | | |
| --- | --- | --- | --- | --- | --- | --- | --- | --- |
| Test | Absolute Sensitivity | Absolute Specificity | Diagnostic OR  [Rank] | Superiority Index [Rank] | Relative Sensitivity | Relative Specificity | Datasets, n | Studies, n |
| 18F-NaF PET/CT | 0.89  (0.66-0.98) | 0.84  (0.56-0.96) | 127.93  (7.53-550.20)  [3] | 2.97  (0.20-9.00)  [2] | 1.00  (1.00-1.00) | 1.00  (1.00-1.00) | 4 | 4 |
| 18F-Choline PET/CT | 0.86  (0.62-0.97) | 0.88  (0.72-0.95) | 92.50  (9.48-359.89)  [4] | 2.53  (0.20-7.00)  [4] | 0.98  (0.69-1.32) | 1.06  (0.83-1.55) | 5 | 5 |
| 68Ga-PSMA PET/CT | 0.77  (0.36-0.98) | 0.88  (0.44-1.00) | 8473793.21  (1.47-463081.1)  [1] | 3.23  (0.11-9.00)  [1] | 0.87  (0.40-1.28) | 1.07  (0.51-1.66) | 2 | 2 |
| 1.5-T MRI | 0.59  (0.24-0.87) | 0.74  (0.33-0.96) | 10.57  (0.44-54.67)  [5] | 0.30  (0.11-1.00)  [5] | 0.67  (0.26-1.03) | 0.89  (0.40-1.43) | 2 | 2 |
| 3.0-T MRI | 0.85  (0.53-0.98) | 0.85  (0.51-0.98) | 175.22  (3.64-1046.39)  [2] | 2.69  (0.14-9.00)  [3] | 0.96  (0.60-1.31) | 1.02  (0.59-1.56) | 4 | 4 |
| Data are reported as mean (range) unless otherwise indicated.  PET/CT=Positron emission tomography/computed tomography; MRI=Magnetic resonance imaging; PCa=Prostate cancer; T=Tesla; NaF=Sodium fluoride; PSMA=Prostate membrane antigen; OR=Odds ratio | | | | | | | | |

| **eTable 28. PET/CT with different tracers and MRI with different numbers of sequences for detecting bone metastasis in patients with PCa by deleting studies with only one diagnostic test** | | | | | | | | |
| --- | --- | --- | --- | --- | --- | --- | --- | --- |
| Test | Absolute Sensitivity | Absolute Specificity | Diagnostic OR  [Rank] | Superiority Index [Rank] | Relative Sensitivity | Relative Specificity | Datasets, n | Studies, n |
| 18F-NaF PET/CT | 0.89  (0.66-0.98) | 0.86  (0.61-0.96) | 135.94  (8.44-621.41)  [3] | 3.35  (0.14-9.00)  [2] | 1.00  (1.00-1.00) | 1.00  (1.00-1.00) | 4 | 4 |
| 18F-Choline PET/CT | 0.88  (0.71-0.96) | 0.88  (0.70-0.96) | 102.26  (11.64-347.79)  [4] | 3.22  (0.14-9.00)  [3] | 1.00  (0.78-1.34) | 1.04  (0.81-1.44) | 6 | 6 |
| 11C-Choline PET/CT | 0.67  (0.21-0.96) | 0.81  (0.30-1.00) | 373.59  (0.47-2413.24)  [2] | 1.63  (0.09-9.00)  [5] | 0.76  (0.23-1.21) | 0.96  (0.35-1.47) | 1 | 1 |
| 68Ga-PSMA PET/CT | 0.78  (0.36-0.99) | 0.88  (0.45-1.00) | 332496.95  (1.86-586917)  [1] | 4.10  (0.11-11.00)  [1] | 0.88  (0.39-1.29) | 1.04  (0.51-1.53) | 2 | 2 |
| Multi-sequence  MRI | 0.85  (0.67-0.96) | 0.87  (0.68-0.96) | 73.18  (8.26-266.72)  [5] | 2.13  (0.14-9.00)  [4] | 0.96  (0.74-1.30) | 1.03  (0.78-1.45) | 7 | 7 |
| Single-sequence MRI | 0.50  (0.10-0.91) | 0.78  (0.29-0.99) | 36.56  (0.19-254.16)  [6] | 0.57  (0.09-5.00)  [6] | 0.57  (0.11-1.05) | 0.93  (0.34-1.42) | 1 | 1 |
| Data are reported as mean (range) unless otherwise indicated.  PET/CT=Positron emission tomography/computed tomography; MRI=Magnetic resonance imaging; PCa=Prostate cancer; NaF=Sodium fluoride; PSMA=Prostate membrane antigen; OR=Odds ratio | | | | | | | | |

| **eTable 29. PET/CT with different tracers and MRI with/without DWI for detecting bone metastasis in patients with PCa by deleting studies with only one diagnostic test** | | | | | | | | |
| --- | --- | --- | --- | --- | --- | --- | --- | --- |
| Test | Absolute Sensitivity | Absolute Specificity | Diagnostic OR  [Rank] | Superiority Index [Rank] | Relative Sensitivity | Relative Specificity | Datasets, n | Studies, n |
| 18F-NaF PET/CT | 0.86  (0.56-0.98) | 0.86  (0.53-0.98) | 157.95  (4.51-837.93)  [5] | 2.56  (0.11-9.00)  [3] | 1.00  (1.00-1.00) | 1.00  (1.00-1.00) | 3 | 3 |
| 18F-Choline PET/CT | 0.87  (0.62-0.97) | 0.87  (0.65-0.97) | 114.82  (8.25-515.65)  [6] | 2.32  (0.14-9.00)  [5] | 1.03  (0.73-1.55) | 1.05  (0.74-1.65) | 5 | 5 |
| 11C-Choline PET/CT | 0.70  (0.21-0.97) | 0.76  (0.25-0.99) | 164.40  (0.39-1380.15)  [4] | 1.20  (0.09-9.00)  [6] | 0.83  (0.24-1.42) | 0.91  (0.29-1.58) | 2 | 2 |
| 68Ga-PSMA PET/CT | 0.77  (0.35-0.99) | 0.88  (0.44-1.00) | 3655902.04  (1.42-636843)  [1] | 3.37  (0.09-11.00)  [1] | 0.91  (0.40-1.48) | 1.05  (0.49-1.74) | 2 | 2 |
| DWI used  MRI | 0.87  (0.55-0.99) | 0.86  (0.54-0.98) | 284.46  (4.91-1807.18)  [3] | 3.10  (0.11-11.00)  [2] | 1.03  (0.63-1.58) | 1.04  (0.61-1.65) | 5 | 5 |
| No-DWI MRI | 0.81  (0.37-0.99) | 0.81  (0.39-0.99) | 420.76  (1.40-2649.15)  [2] | 2.41  (0.09-11.00)  [4] | 0.97  (0.44-1.57) | 0.97  (0.44-1.61) | 2 | 2 |
| Data are reported as mean (range) unless otherwise indicated.  PET/CT=Positron emission tomography/computed tomography; MRI=Magnetic resonance imaging; PCa=Prostate cancer; DWI=Diffusion weighted imaging; NaF=Sodium fluoride; PSMA=Prostate membrane antigen; OR=Odds ratio | | | | | | | | |

| **eTable 30. PET/CT with different tracers and MRI with different numbers of imaging planes for detecting bone metastasis in patients with PCa by deleting studies with only one diagnostic test** | | | | | | | | |
| --- | --- | --- | --- | --- | --- | --- | --- | --- |
| Test | Absolute Sensitivity | Absolute Specificity | Diagnostic OR  [Rank] | Superiority Index [Rank] | Relative Sensitivity | Relative Specificity | Datasets, n | Studies, n |
| 18F-NaF PET/CT | 0.89  (0.64-0.98) | 0.85  (0.59-0.96) | 137.36  (7.28-615.56)  [4] | 2.88  (0.14-9.00)  [4] | 1.00  (1.00-1.00) | 1.00  (1.00-1.00) | 4 | 4 |
| 18F-Choline PET/CT | 0.88  (0.70-0.97) | 0.88  (0.71-0.96) | 105.10  (13.13-366.63)  [5] | 2.94  (0.20-9.00)  [3] | 1.00  (0.77-1.38) | 1.06  (0.81-1.52) | 6 | 6 |
| 11C-Choline PET/CT | 0.65  (0.19-0.96) | 0.80  (0.29-1.00) | 355.70  (0.39-2081.50)  [2] | 1.38  (0.09-9.00)  [5] | 0.73  (0.22-1.18) | 0.97  (0.34-1.54) | 1 | 1 |
| 68Ga-PSMA PET/CT | 0.76  (0.36-0.99) | 0.90  (0.45-1.00) | 331207.58  (1.45-625923.72)  [1] | 3.98  (0.11-11.00)  [1] | 0.87  (0.39-1.32) | 1.08  (0.53-1.62) | 2 | 2 |
| 1 imaging plane  MRI | 0.87  (0.58-0.99) | 0.87  (0.63-0.97) | 231.86  (6.87-1240.51)  [3] | 3.38  (0.14-9.00)  [2] | 0.99  (0.64-1.38) | 1.05  (0.74-1.53) | 5 | 5 |
| ≥2 imaging planes MRI | 0.67  (0.37-0.88) | 0.81  (0.45-0.96) | 19.84  (1.24-85.46)  [6] | 0.50  (0.09-3.00)  [6] | 0.76  (0.41-1.13) | 0.97  (0.52-1.43) | 3 | 3 |
| Data are reported as mean (range) unless otherwise indicated.  PET/CT=Positron emission tomography/computed tomography; MRI=Magnetic resonance imaging; PCa=Prostate cancer; NaF=Sodium fluoride; PSMA=Prostate membrane antigen; OR=Odds ratio | | | | | | | | |

| **eTable 31. PET/CT with different tracers and MRI with different coverage for detecting bone metastasis in patients with PCa by deleting studies with only one diagnostic test** | | | | | | | | |
| --- | --- | --- | --- | --- | --- | --- | --- | --- |
| Test | Absolute Sensitivity | Absolute Specificity | Diagnostic OR  [Rank] | Superiority Index [Rank] | Relative Sensitivity | Relative Specificity | Datasets, n | Studies, n |
| 18F-NaF PET/CT | 0.89  (0.67-0.98) | 0.85  (0.60-0.96) | 135.77  (8.05-626.54)  [4] | 2.89  (0.14-9.00)  [2] | 1.00  (1.00-1.00) | 1.00  (1.00-1.00) | 4 | 4 |
| 18F-Choline PET/CT | 0.86  (0.65-0.96) | 0.89  (0.72-0.96) | 98.50  (10.30-340.56)  [5] | 2.67  (0.14-9.00)  [4] | 0.98  (0.72-1.29) | 1.06  (0.82-1.50) | 6 | 6 |
| 11C-Choline PET/CT | 0.75  (0.34-0.95) | 0.92  (0.56-1.00) | 252.10  (2.70-1475.24)  [2] | 2.70  (0.11-9.00)  [3] | 0.84  (0.39-1.22) | 1.10  (0.65-1.59) | 3 | 3 |
| 68Ga-PSMA PET/CT | 0.79  (0.37-0.99) | 0.89  (0.45-1.00) | 803620.51  (1.51-920665)  [1] | 4.29  (0.09-11.00)  [1] | 0.89  (0.41-1.29) | 1.06  (0.52-1.57) | 2 | 2 |
| Axial skeleton or WB MRI | 0.76  (0.54-0.91) | 0.88  (0.73-0.95) | 36.52  (5.97-120.63)  [6] | 0.91  (0.11-5.00)  [6] | 0.86  (0.60-1.16) | 1.05  (0.84-1.50) | 8 | 8 |
| Pelvis MRI | 0.70  (0.23-0.98) | 0.76  (0.27-0.99) | 140.59  (0.39-873.03)  [3] | 1.24  (0.09-9.00)  [5] | 0.79  (0.25-1.24) | 0.91  (0.31-1.41) | 1 | 1 |
| Data are reported as mean (range) unless otherwise indicated.  PET/CT=Positron emission tomography/computed tomography; MRI=Magnetic resonance imaging; PCa=Prostate cancer; NaF=Sodium fluoride; PSMA=Prostate membrane antigen; WB=Whole body; OR=Odds ratio | | | | | | | | |

| **eTable 32. PET/CT with different tracers and 3.0-T high-quality MRI for detecting bone metastasis in patients with PCa by deleting studies with only one diagnostic test** | | | | | | | | |
| --- | --- | --- | --- | --- | --- | --- | --- | --- |
| Test | Absolute Sensitivity | Absolute Specificity | Diagnostic OR  [Rank] | Superiority Index [Rank] | Relative Sensitivity | Relative Specificity | Datasets, n | Studies, n |
| 18F-NaF PET/CT | 0.82  (0.47-0.97) | 0.85  (0.46-0.98) | 123.46  (2.94-643.83)  [3] | 1.29  (0.14-5.00)  [3] | 1.00  (1.00-1.00) | 1.00  (1.00-1.00) | 3 | 3 |
| 18F-Choline PET/CT | 0.84  (0.51-0.98) | 0.84  (0.59-0.95) | 95.97  (4.24-521.15)  [4] | 1.18  (0.14-5.00)  [4] | 1.04  (0.63-1.66) | 1.03  (0.70-1.79) | 4 | 4 |
| 68Ga-PSMA PET/CT | 0.75  (0.34-0.98) | 0.89  (0.43-1.00) | 1462949.17  (1.33-422581.01)  [2] | 2.01  (0.14-7.00)  [2] | 0.94  (0.41-1.56) | 1.09  (0.49-1.99) | 2 | 2 |
| 3.0-T high-quality MRI | 0.89  (0.50-1.00) | 0.86  (0.48-1.00) | 8688299.84  (3.36-943665.57)  [1] | 3.17  (0.14-7.00)  [1] | 1.12  (0.59-1.86) | 1.06  (0.55-1.91) | 3 | 3 |
| Data are reported as mean (range) unless otherwise indicated.  High-quality MRI was referred to the MRI equipped with multi-sequence, DWI used and ≥2 imaging planes.  PET/CT=Positron emission tomography/computed tomography; MRI=Magnetic resonance imaging; PCa=Prostate cancer; NaF=Sodium fluoride; PSMA=Prostate membrane antigen; T=Tesla; DWI=Diffusion weighted imaging; OR=Odds ratio | | | | | | | | |

| **eTable 33. PET/CT with different tracers and MRI for detecting bone metastasis in patients with PCa on lesion-based level** | | | | | | | | |
| --- | --- | --- | --- | --- | --- | --- | --- | --- |
| Test | Absolute Sensitivity | Absolute Specificity | Diagnostic OR  [Rank] | Superiority Index [Rank] | Relative Sensitivity | Relative Specificity | Datasets, n | Studies, n |
| 18F-NaF PET/CT | 0.83  (0.73-0.93) | 0.73  (0.60-0.86) | 30.96  (2.23-134.75)  [5] | 1.24  (0.11-5.00)  [5] | 1.00  (1.00-1.00) | 1.00  (1.00-1.00) | 5 | 3 |
| 18F-Choline PET/CT | 0.76  (0.68-0.84) | 0.91  (0.83-0.99) | 66.63  (6.35-237.76)  [3] | 1.79  (0.14-7.00)  [3] | 0.93  (0.77-1.09) | 1.29  (1.00-1.58) | 5 | 4 |
| 11C-Choline PET/CT | 0.84  (0.70-0.98) | 0.78  (0.57-0.99) | 348.36  (1.25-2479.33)  [2] | 2.92  (0.11-9.00)  [1] | 1.03  (0.79-1.27) | 1.11  (0.72-1.50) | 2 | 2 |
| 68Ga-PSMA PET/CT | 0.78  (0.57-0.99) | 0.79  (0.57-1.01) | 106887.31  (0.55-191176.67)  [1] | 2.90  (0.11-9.00)  [2] | 0.96  (0.65-1.27) | 1.11  (0.71-1.51) | 1 | 1 |
| MRI | 0.80  (0.69-0.91) | 0.85  (0.75-0.95) | 50.64  (3.58-212.23)  [4] | 1.65  (0.14-7.00)  [4] | 0.97  (0.76-1.18) | 1.20  (0.90-1.50) | 5 | 5 |
| Data are reported as mean (range) unless otherwise indicated.  PET/CT=Positron emission tomography/computed tomography; MRI=Magnetic resonance imaging; PCa=Prostate cancer; NaF=Sodium fluoride; PSMA=Prostate membrane antigen; OR=Odds ratio | | | | | | | | |

| **eTable 34. PET/CT with different tracers and 1.5-T high-quality MRI for detecting bone metastasis in patients with PCa on lesion-based level** | | | | | | | | |
| --- | --- | --- | --- | --- | --- | --- | --- | --- |
| Test | Absolute Sensitivity | Absolute Specificity | Diagnostic OR  [Rank] | Superiority Index [Rank] | Relative Sensitivity | Relative Specificity | Datasets, n | Studies, n |
| 18F-NaF PET/CT | 0.84  (0.74-0.94) | 0.73  (0.60-0.86) | 30.68  (2.35-124.15)  [5] | 1.31  (0.14-5.00)  [5] | 1.00  (1.00-1.00) | 1.00  (1.00-1.00) | 5 | 3 |
| 18F-Choline PET/CT | 0.77  (0.69-0.85) | 0.90  (0.82-0.98) | 68.17  (6.27-259.92)  [3] | 1.93  (0.14-7.00)  [3] | 0.93  (0.78-1.08) | 1.28  (1.00-1.56) | 5 | 4 |
| 11C-Choline PET/CT | 0.82  (0.66-0.98) | 0.79  (0.58-1.00) | 314.76  (1.18-2149.95)  [2] | 2.73  (0.11-9.00)  [2] | 0.99  (0.74-1.24) | 1.13  (0.75-1.51) | 2 | 2 |
| 68Ga-PSMA PET/CT | 0.77  (0.55-0.99) | 0.80  (0.59-1.01) | 173644.23  (0.53-165321.57)  [1] | 2.95  (0.11-9.00)  [1] | 0.93  (0.63-1.23) | 1.13  (0.73-1.53) | 1 | 1 |
| 1.5-T high-quality MRI | 0.79  (0.64-0.94) | 0.80  (0.67-0.93) | 66.45  (1.85-387.92)  [4] | 1.74  (0.11-7.00)  [4] | 0.96  (0.72-1.20) | 1.14  (0.83-1.45) | 3 | 3 |
| Data are reported as mean (range) unless otherwise indicated.  High-quality MRI was referred to the MRI equipped with multi-sequence, DWI used, and ≥2 imaging planes.  PET/CT=Positron emission tomography/computed tomography; MRI=Magnetic resonance imaging; PCa=Prostate cancer; NaF=Sodium fluoride; PSMA=Prostate membrane antigen; T=Tesla; DWI=Diffusion weighted imaging; OR=Odds ratio | | | | | | | | |
